# Metallacarborane Complex Boosts the Rate of DNA Oligonucleotide Hydrolysis in the Reaction Catalyzed by Snake Venom Phosphodiesterase

**DOI:** 10.3390/biom10050718

**Published:** 2020-05-05

**Authors:** Damian Kaniowski, Katarzyna Kulik, Katarzyna Ebenryter-Olbińska, Ewelina Wielgus, Zbigniew Lesnikowski, Barbara Nawrot

**Affiliations:** 1 Centre of Molecular and Macromolecular Studies, Polish Academy of Sciences, Sienkiewicza 112, 90-363 Lodz, Poland; dkanio@cbmm.lodz.pl (D.K.); kpieta@cbmm.lodz.pl (K.K.); kebenryt@cbmm.lodz.pl (K.E.-O.); ms@cbmm.lodz.pl (E.W.); 2 Institute of Medical Biology, Polish Academy of Sciences, Laboratory of Medicinal Chemistry, 106 Lodowa St., 92-232 Lodz, Poland; zlesnikowski@cbm.pan.pl

**Keywords:** antisense oligonucleotide, metallacarborane, boron cluster, snake venom, phosphodiesterase, exonuclease, BNCT, binding affinity, MST

## Abstract

Antisense oligonucleotides conjugated with boron clusters (B-ASOs) have been described as potential gene expression inhibitors and carriers of boron for boron neutron capture therapy (BNCT), providing a dual-action therapeutic platform. In this study, we tested the nucleolytic stability of DNA oligonucleotides labeled with metallacarborane [(3,3’-iron-1,2,1’,2’-dicarbollide)(−1)]ate [Fe(C_2_B_9_H_11_)_2_] (FESAN) against snake venom phosphodiesterase (svPDE, 3’→5’-exonuclease). Contrary to the previously observed protective effect of carborane (C_2_B_10_H_12_) modifications, the B-ASOs containing a metallacarborane moiety at the 5’-end of the oligonucleotide chain were hydrolyzed faster than their parent nonmodified oligomers. Interestingly, an enhancement in the hydrolysis rate was also observed in the presence of free metallacarborane, and this reaction was dependent on the concentration of the metallacarborane. Microscale thermophoresis (MST) analysis confirmed the high affinity (*K*_d_ nM range) of the binding of the metallacarborane to the proteins of crude snake venom and the moderate affinity (*K*_d_ µM range) between the metallacarborane and the short single-stranded DNA. We hypothesize that the metallacarborane complex covalently bound to B-ASO holds DNA molecules close to the protein surface, facilitating enzymatic cleavage. The addition of metallacarborane alone to the ASO/svPDE reaction mixture provides the interface to attract freely floating DNA molecules. In both cases, the local DNA concentration around the enzymes increases, giving rise to faster hydrolysis. It was experimentally shown that an allosteric effect, possibly attributable to the observed boost in the 3’→5’-exonucleolytic activity of snake venom phosphodiesterase, is much less plausible.

## 1. Introduction

**Scheme 1:**
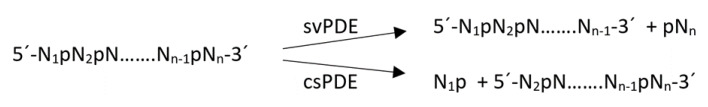
Products of the cleavage of DNA/RNA oligonucleotides catalyzed by 3’→5’-exonuclease (svPDE) and 5’→3’-exonuclease (csPDE). N is the nucleoside unit, and p is the abbreviation for the phosphate group.

The inhibition of gene expression via the antisense approach involves the use of a DNA oligonucleotide (typically 13–25 mers) to “arrest” the target RNA by hybridization according to the Watson-Crick mode followed by the cleavage of mRNA in the resultant heteroduplex by RNase H [1,2]. Some antisense oligonucleotides (ASOs) directed towards the 5’-end of the target mRNA or to the region of the AUG start codon are designed to inhibit mRNA function by sterically blocking the translational machinery [3], but other mechanisms may be used to interfere with the mRNA maturation process [4], including inhibition of the 5’-cap formation [5], inhibition of the RNA splicing [6], blocking of the pre-mRNA polyadenylation [7], or translation termination without RNA degradation [8,9]. Unfortunately, natural ASOs are readily hydrolyzed by nucleases present in body fluids [10]; thus, a plethora of nucleic acid analogs have been generated in hopes of preserving or enhancing their ability to hybridize with mRNA and improving their stability in vivo [11,12]. Increased resistance to nucleases is one of the most preferred and desirable features, and usually such stability tests are executed very early in the evaluation of new analogs. These tests are often performed using 3’- and 5’-exonucleases, typically with snake venom phosphodiesterase (svPDE, also called 5’-phosphodiesterase), which hydrolyses oligonucleotide substrates in the 3’→5’ direction [13], and calf spleen phosphodiesterase (csPDE), which proceeds in the opposite (5’→3’) direction (Scheme 1) [14].

The 5’-phosphodiesterase present in crude venom from *Crotalus atrox* has remarkable stability and accepts a wide spectrum of substrates [15]. Nonetheless, its nucleolytic activity towards phosphorothioate (PS), locked nucleic acids (LNA), 2’-deoxy-2’-fluoro-beta-D-arabinonucleic acid (FANA), and 2’-*O*-methyl- (2’-OMe) or 2’-*O*-methoxyethyl- (2’-MOE) DNA and RNA analogs or oligomers with an unnatural phosphate backbone, such as phosphate morpholino and phosphorodiamidate morpholino (PMO) and peptide nucleic acid (PNA) constructs, is either partially reduced or fully inhibited [16,17]. To capitalize on the good hybridizing properties of oligonucleotides otherwise susceptible to nucleases, tracks several nucleotides long are often flanked at the 3’- and 5’-ends with several modified units, which provide these so-called *gapmers* with some resistance to cellular endo- and exonucleases [18].

Recently, several antisense oligonucleotides containing boron clusters (B-ASOs) were reported to inhibit the biosynthesis of the epidermal growth factor receptor (EGFR) [19,20,21] and the beta-secretase (BACE1) protein [22]. B-ASOs may exert dual actions, because in addition to antisense activity, they offer a therapeutic platform for boron neutron capture therapy (BNCT) [23]. In BNCT, ^10^B boron atoms are delivered to tumor cells, and upon irradiation with a neutron beam, they absorb neutrons and become ^11^B. They are highly unstable and split into the high-linear energy transfer (LET) ^4^He and ^7^Li species, which kill the cancer cells in which they are generated. Because previous studies on B-ASOs, including susceptibility to enzymatic degradation, were focused mainly on carborane-modified ASOs [24,25,26] and to a lesser extent, on metallacarborane modifications [27,28,29] and because of potential medical applications of metallacarborane-modified ASOs, we decided to study the action of snake venom phosphodiesterase on modified DNA oligomers in more detail. We have found that contrary to carborane modification, which usually increases oligonucleotide stability in the presence of nucleolytic enzymes, ferra(III) bis(dicarbollide) modification has the opposite effect. The results of this study, the proposed mechanism of the observed phenomenon and its potential practical advantages are the subject of the present communication.

## 2. Materials and Methods

### 2.1. Chemistry

Unmodified nucleoside phosphoramidites and 5’-dimethoxytrityl-2’-propargyluridine 3’-*O*-(*N*,*N*-diisopropyl-2-cyanoethyl) phosphoramidite were purchased from ChemGenes Corporation (Wilmington, MA, USA). [(3,3’-Iron-1,2,1’,2’-dicarbollide)(−1)]ate (-1) cesium salt (ferra(III) bis(dicarbollide), FESAN) was purchased from Katchem (Režn/Prague, Czech Republic). Radio-labeled adenosine 5’-triphosphate ([γ-^32^P]-ATP) was purchased from Hartmann-Analytik (Braunschweig, Germany), and T4 polynucleotide kinase and kinase buffer were purchased from BioLabs (New England, MA, USA). Autoradiography double-coated films were purchased from Carestream Medical X-ray blue (MXBE film, Rochester, NY, USA). The developer reagent and fixer reagent were from Kodak Processing Chemicals (Sigma Aldrich, St. Louis, MO, USA). The snake venom phosphodiesterase I from *Crotalus atrox* Western Diamondback Rattlesnake was purchased from Sigma-Aldrich (USA).

#### 2.1.1. Automated Synthesis of Oligonucleotides

The alkyne-functionalized DNA oligonucleotides were synthesized according to the phosphoramidite solid-phase approach using a LCA CPG solid support and commercially available phosphoramidites of T, dC, dA, dG and 2’-*O*-propargyl-5’-DMT-uridine with DMT protecting groups at their 5’-hydroxy moieties, and their exocyclic amine functions were protected with acetyl, benzoyl and *iso*-butyryl groups for dC, dA and dG, respectively. The commercial nucleoside-linked LCA-CPG solid support and 0.07 M solutions of the monomers in CH_3_CN were used. The oligonucleotides were synthesized on a 0.1 µmol scale using an H6 GeneWorld automated DNA/RNA synthesizer (K&A, Laborgeraete GbR, Schaafheim, Germany) under the standard conditions for DNA oligonucleotide synthesis [30]. All compounds were cleaved from the solid support as 5’-DMT-protected derivatives and purified by RP-HPLC (“DMT-on” mode). Deprotection of the 5’-OH groups was performed on C18 SepPak cartridges (Waters, Milford, Ireland) with 2% TFA. The sequence and purity of the obtained compounds were confirmed by MALDI-TOF or ESI-Q-TOF mass spectrometry, analytical RP-HPLC and denaturing polyacrylamide gel electrophoresis (PAGE, 7 M urea). Nonmodified oligonucleotides **1** and **2** and their 5’-fluorescently labeled derivatives were obtained by an analogous method using a routine DNA oligonucleotide synthesis process. The fluorescein phosphoramidite monomer was obtained from Glen Research. The RP-HPLC chromatograms and mass spectra of **1**, **2**, **P1a**–**P1c**, **P2a**, **P2b**, **FL-1** and **FL-2** oligomers are given in Figure S1 in the Supplementary Material.

#### 2.1.2. Synthesis of Oligonucleotides **1a**, **1b**, **1c** and **2a**, **2b** Modified with Metallacarborane

**Scheme 2:**
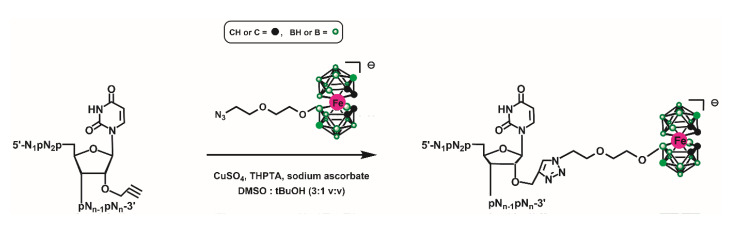
Labeling of oligonucleotides **1** and **2** with 3-iron-bis(1,2-dicarbollide)ate, a metallacarborane moiety bearing an Fe^3+^ cation.

Oligonucleotides **P1a**, **P1b**, **P1c**, **P2a** and **P2b** were post-synthetically modified with boron clusters containing negatively charged (3,3’-iron-1,2,1’,2’-dicarbollide)(-1)]ate linked with a 5-azido-3-oxa-pentoxy group (-OCH_2_CH_2_OCH_2_CH_2_N_3_) [29] at the 8 position by a copper(I)-catalyzed azide-alkyne cycloaddition reaction. The reactions were performed according to the standard copper sulfate procedure used in the chemistry of oligonucleotides [31]. First, the following solutions were prepared: (1) DMSO/tBuOH (3:1 *v*/*v*), (2) 0.1 M copper sulfate in water, (3) 0.2 M tris(3-hydroxypropyltriazolylmethyl) amine (THPTA) in DMSO/tBuOH and (4) 520 mM ferra(III) bis(dicarbollide)-8-alkyl azide (Scheme 2) in DMSO/tBuOH. Then, the THPTA and copper sulfate solutions were mixed at a 1:1 ratio and degassed in an argon stream. Fifty molar equivalents of metallacarborane-alkyl azide (3.34 µL) and 25 molar equivalents of the copper sulfate/THPTA solution (9.19 µL) were added to a vial containing the oligonucleotide (4 OD) in water (40 µL). The mixture was vortexed, and if a precipitate formed, ca. 80 µL of DMSO/tBuOH (3:1, *v*/*v*) was added. Afterwards, 40 equivalents of freshly prepared 0.1 M sodium ascorbate in water (14.4 µL) was added to the reaction mixture. The final solution was briefly degassed with an argon stream and agitated at room temperature for 4 h. Then, the products were isolated by RP-HPLC using a Kinetex 5 µm, C18, 250 × 4.6 mm column with buffer A (0.1 M CH_3_COONH_4_/H_2_O) and buffer B (100% CH_3_CN) solvent system at a flow rate of 1 mL/min. The buffer gradient was as follows: (1) 0–2 min 0% B; (2) 2–25 min 0%–48% B; (3) 25–30 min 48%–60% B; (4) 30–35 min 60%–0% B; and (5) 35–38 min 0% B. UV detection was performed at λ_max_ = 268 nm. The collected oligonucleotide fractions were desalted on C18 SepPak cartridges (Waters). The molecular mass of each synthesized oligonucleotide was confirmed by MALDI-TOF or ESI-Q-TOF mass spectrometry (Table 1, Figure S1), and the purity was confirmed by polyacrylamide gel electrophoresis (PAGE, Figure S2).

### 2.2. Enzymatic Assays

#### 2.2.1. 5’-Phosphorylation of Oligonucleotides

A 0.10 OD ODN (0.91 nmol of **1** or 0.75 nmol of **2**) solution in 15 µL of Milli-Q water was mixed with ^32^P-radiolabeled ATP (37.0 MBq, 1.00 mCi, diluted 10x with Milli-Q water, 2 µL), T4 polynucleotide kinase (1 µL, 10.000 unit/mL) and 2 µL kinase buffer supplied by the manufacturer. The reaction mixture, which had a total volume of 20 µL, was incubated at 37 °C for 1 h. Next, the mixture was inactivated by incubation for 3 min at 80 °C and used in further studies without further purification.

#### 2.2.2. Hydrolysis of Oligonucleotides **1**, **2**, **1a**, **1b**, **1c**, **2a** and **2b** in the Presence of svPDE Analyzed by PAGE

The stability of these oligonucleotides against 3’-exonuclease-mediated degradation was determined using snake venom phosphodiesterase I from *Crotalus atrox* Western Diamondback Rattlesnake (crude dried venom, vial of ≥0.01 units/mg solid, type IV). The enzyme was dissolved in water according to the manufacturer’s protocol. The ^32^P-radiolabeled oligonucleotide (0.02 OD, 3 µL of stock solution after phosphorylation) was mixed with Milli-Q water (21 µL) and reaction buffer (3 µL of 150 mM MgCl_2_, pH 9) at 4 °C, and then the snake venom phosphodiesterase (3 µL of 0.5 mU/µL stock solution) was added. The total volume was 30 µL. The resultant assay mixture was incubated at 37 °C for up to 90 min. Aliquots of the enzymatic reaction (4 µL) mixture were withdrawn from the reaction mixture at predetermined times (0, 5, 15, 30, 60 and 90 min) and mixed with loading buffer (10 mM Tris-HCl, 60 mM EDTA, 60% glycerol, 0.03% bromophenol blue, 0.03% cyanol, pH 7.6) (6 µL), and the mixture was inactivated by incubation for 3 min at 80 °C. Each sample was analyzed by 20% gel denaturing polyacrylamide gel electrophoresis (PAGE) with 7 M urea at room temperature at 20 mA for 2 h. After electrophoresis was complete, the gel was transferred to an exposure cassette and covered with autoradiography double-coated films for 10 min at low temperature (−25 °C). Then, the double-coated film was soaked in the developing reagent and then in the fixing reagent and scanned using a G-Box apparatus (Syngene, Cambridge, UK).

For the analogous analyses of the svPDE-catalyzed hydrolysis of **1** and **2** in the presence of ferra(III) bis(dicarbollide), the ^32^P-oligonucleotide (0.02 OD, 3 µL of stock solution after phosphorylation) was mixed at 4 °C with a solution of the boron cluster in Milli-Q water (182 nM, 21 µL) and with the reaction buffer (3 µL of 150 mM MgCl_2_, pH 9) at 4 °C and then the snake venom phosphodiesterase (3 µL of 0.5 mU/µL stock solution) was added, and the products of the hydrolysis reaction were analyzed by PAGE, as described above.

#### 2.2.3. Hydrolysis of Oligonucleotides **1**, **2**, **1a**, **1b**, **1c**, **2a** and **2b** in the Presence of svPDE Analyzed by MALDI-TOF MS

Samples of the DNA oligonucleotides (0.1 OD in 9 μL of Milli-Q water containing 15 mM MgCl_2_) were mixed with svPDE (1 μL, 0.5 mU) and incubated at 37 °C. After 0, 5, 15, 30, 60 and 90 min, 1 μL aliquots were withdrawn, mixed with 1 μL of the matrix and applied directly to the sample plate. As the matrix, a mixture of 50 mg/mL 3-hydroxypicolinic acid in 50% acetonitrile and 50 mg/mL diammonium hydrogen citrate in deionized water (8:1, *v*/*v*) was used. After allowing the samples to dry and crystallize for a few minutes, the samples were analyzed by MALDI-TOF mass spectrometry. The MALDI-TOF mass spectra of all the synthesized compounds as well as the products of the svPDE-assisted hydrolysis were recorded in negative mode on an Axima Performance (Shimadzu) instrument equipped with a nitrogen laser (337 nm) in linear mode. Each mass spectrum was accumulated from at least 200 laser shots and processed by Biotech Launchpad ver. 2.9.1 program (Shimadzu).

For the analogous analyses of the svPDE-catalyzed hydrolysis of **1** and **2** in the presence of ferra(III) bis(dicarbollide), the oligonucleotide (0.1 OD) in 8 μL of Milli-Q water was mixed with the boron clusters (0, 91, 182 or 273 nM), MgCl_2_ (1 µL, 150 mM) and svPDE (1 μL, 0.5 mU) and incubated at 37 °C. After 0, 5, 15, 30, 60 and 90 min, 1 μL aliquots were withdrawn, mixed with 1 μL of the matrix (3-hydroxypicolinic acid-diammonium hydrogen citrate) and applied directly to the sample plate. The products of the hydrolysis were analyzed by MALDI-TOF MS, as described above.

### 2.3. Affinity Measurements

#### 2.3.1. Affinity of Ferra(III) bis(dicarbollide) for Crude *Crotalus Atrox* Venom Analyzed by MST

Microscale thermophoresis (MST) analysis was performed using a NanoTemper Monolith NT.115 instrument (NanoTemper Technologies GmbH). Snake venom phosphodiesterase I from *Crotalus atrox* Western Diamondback Rattlesnake (crude dried venom) at a concentration of 0.5 mU was fluorescently labeled with a RED-NHS 2nd generation fluorescent dye labeling kit (NanoTemper Technologies GmbH) according to the manufacturer’s instructions and using seawater solution supplemented with 0.05% Tween. Varying concentrations of ferra(III) bis(dicarbollide) (182 pM to 3000 nM) were titrated against labeled enzyme dissolved in MST buffer (50 mM Tris-HCl, pH 7.5, 10 mM MgCl_2_, 150 mM NaCl) supplemented with 0.05% Tween. Samples were loaded into premium-coated capillaries (NanoTemper Technologies GmbH), and MST measurements were performed using 20% MST power and 90% light-emitting diode (LED) power. The laser on and off times were 30 and 5 s, respectively. A set of MST binding experiments conducted at two different temperatures were carried out at 630 nm. The datasets were processed with MO Affinity Analysis software (NanoTemper Technologies GmbH) using the signal from the initial fluorescence.

#### 2.3.2. Affinity of Oligonucleotides **FL-1** and **FL-2** to Ferra(III) bis(dicarbollide) Analyzed by MST

5’-Fluorescein-labeled oligonucleotide **FL-1** or **FL-2** (10 nmol/L solution in MST buffer supplemented with 0.05% Tween) was titrated with ferra(III) bis(dicarbollide) (182 pM to 3000 nM) dissolved in the same buffer, as described above. Samples were loaded into standard capillaries (NanoTemper Technologies GmbH), and MST measurements were performed using 20% MST power and 90% LED power. The laser on and off times were 30 and 5 s, respectively. For each set of binding experiments, three independent MST measurements were carried out at 495 nm. The datasets were processed with MO Affinity Analysis software (NanoTemper Technologies GmbH) using the signal from the initial fluorescence.

## 3. Results

### 3.1. Chemical Synthesis of Metallacarborane-Conjugated ASO Models

Two reference 13-nt oligonucleotides, **1** and **2** (a homopyrimidine and a purine-enriched oligomer, respectively, Table 1), were designed to be *iso*-sequential with two fragments of a previously reported 22-nt antisense oligonucleotide (ASO-22) directed towards the mRNA of the epidermal growth factor receptor (EGFR) [19,20,21]. Three congeners of **1** (**P1a**, **P1b,** and **P1c**) and two congeners of **2** (**P2a** and **P2b**, Table 1) bearing a 2’-*O*-propargyl uridine (**U_Pr_**) unit were obtained by automated solid-phase synthesis. The propargyl groups were post-synthetically reacted with an alkyl azide moiety present in a linker bearing negatively charged 3,3’-iron-bis(1,2-dicarbollide)ate (a metallacarborane moiety bearing an Fe^3+^ cation, ferra(III) bis(dicarbollide) linked with -OCH_2_CH_2_OCH_2_CH_2_N_3_, via a “click” reaction. Resulting oligonucleotides **1a**, **1b**, **1c**, **2a** and **2b** containing a uridine unit labeled with ferra(III) bis(dicarbollide) (in Table 1 marked as **U_B_**) were isolated by RP-HPLC and characterized by MALDI-TOF or electrospray ionization (ESI) mass spectrometry and electrophoresis in 20% polyacrylamide/7 M gel (PAGE) (Table 1, Figures S1 and S2).

Oligonucleotides **1a** and **1b** possessed **U_B_** units instead of the thymidine residues present in **1** at the 1 or 11 positions, respectively. Because **2** has only one thymidine residue (at position 2), in addition to the corresponding **U_B_**-containing oligomer **2a**, oligomer **2b** was prepared, in which the 2’-deoxycytidine moiety at the 11 position was replaced with a **U_B_** unit.

### 3.2. Hydrolysis of Reference Oligonucleotides ***1*** and ***2*** in the Presence of svPDE Monitored by MALDI-TOF Mass Spectrometry

**Figure 1:**
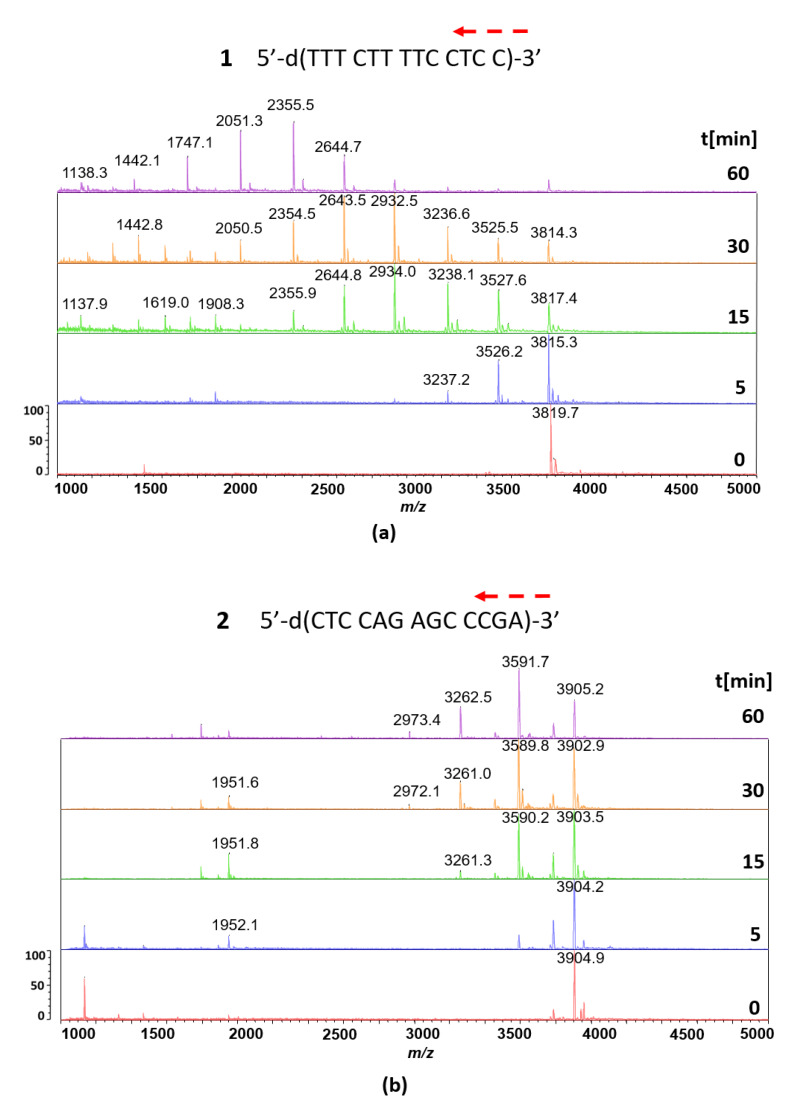
MALDI-TOF mass spectra of the reaction mixtures of the 3’→5’ exonucleolytic hydrolysis of oligonucleotides **1** (**a**) and **2** (**b**) in the presence of svPDE. Samples were analyzed at 0, 5, 15, 30 and 60 min.

**Figure 2:**
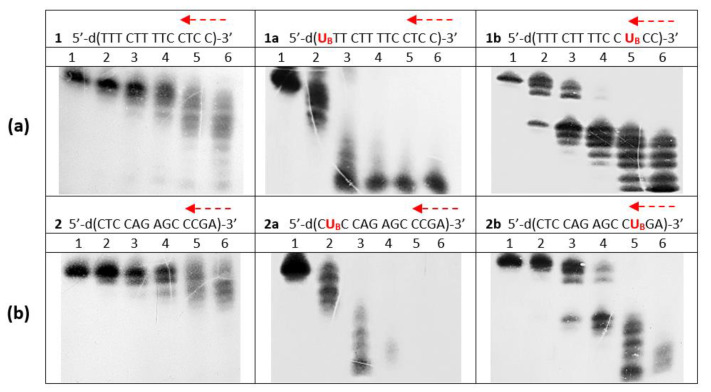
Autoradiograms of 20% denaturing PAGE, showing the cleavage kinetics of 5’-^32^P-labeled oligonucleotides **1**, **1a** and **1b** (panel **a**) and **2**, **2a** and **2b** (panel **b**) after treatment with svPDE over 0–90 min. Lanes 1, 2, 3, 4, 5 and 6 correspond to samples analyzed at 0, 5, 15, 30, 60 and 90 min. The experimental conditions are described in the Materials and Methods section.

Oligonucleotides **1** and **2** were incubated with svPDE in water containing 15 mM MgCl_2_ as recommended by the manufacturer. Magnesium ions were required for the catalytic activity of the svPDE enzyme (see Figure S3). The compositions of the reaction mixtures were determined at 0, 5, 15, 30 and 60 min by MALDI-TOF mass spectrometry (Figure 1). It was found that **1** was hydrolyzed faster than **2**, and after incubation for 60 min, only a small amount of intact **1** remained in the mixture, and its 6-9 nt remnants (detected at *m/z* 1747.1, 2051.3, 2355.5 and 2644.7, respectively) were the main products. At the same time, ca. 25% of **2** (*m/z* 3905) remained intact, and the mixture contained mainly the products truncated by 1 or 2 nucleotides (at *m/z* 3591.7 and 3262.5, respectively). In an independent experiment, **1** and **2** enzymatically labeled at the 5’-end with a radioactive ^32^P-phosphate group were subjected to analogous enzymatic hydrolysis followed by PAGE analysis in a 20% polyacrylamide denaturing gel (7 M urea, also after 90 min, Figure 2a,b). The results of densitometry analysis were in good agreement with the MS data and demonstrated the higher activity of svPDE towards polypyrimidine tracts than purine-enriched tracts in DNA oligonucleotides. Interestingly, this result is not supported by the previously reported data that phosphodiesterases act on native substrates such as DNA, rRNA, and tRNA without showing any preference for purine or pyrimidine bases [15].

### 3.3. svPDE-Assisted Hydrolysis of Metallacarborane-Conjugated Oligonucleotides ***1a***, ***1b***, ***1c***, ***2a***, and ***2b***

For the initial studies, B-ASOs **1a**, **1b**, **2a** and **2b** were enzymatically labeled at the 5’-end with a radioactive ^32^P-phosphate group and treated with svPDE under the same conditions as unmodified **1** and **2**. Surprisingly, PAGE analysis (20% polyacrylamide/7 M urea) of the resultant mixtures showed that **1a** and **2a** were almost completely degraded in 15 min (Figure 2). These results show that the presence of a single **U_B_** unit at the 5’-end significantly accelerated the enzymatic hydrolysis of B-ASOs. Such an acceleration in the svPDE-assisted hydrolysis was also observed for **1b**, where **U_B_** at the 11 position from the 5’-end accelerated at least the first two cleavage steps (Figure 2), because contrary to the hydrolysis of **1**, the substrate had been completely consumed by the 15 min time point despite strong overexposure of the lanes associated with incubation times ≥ 15 min. Notably, in the lane corresponding to 15 min of hydrolysis, a gap between the third and fourth bands from the top indicates the increased mobility of the 10-nt product in the gel after cleavage of the relatively bulky **U_B_** unit, and this feature was confirmed in an independent experiment (see Figure S2). An analogous acceleration following cleavage was observed with **2b** (Figure 2).

**Figure 3:**
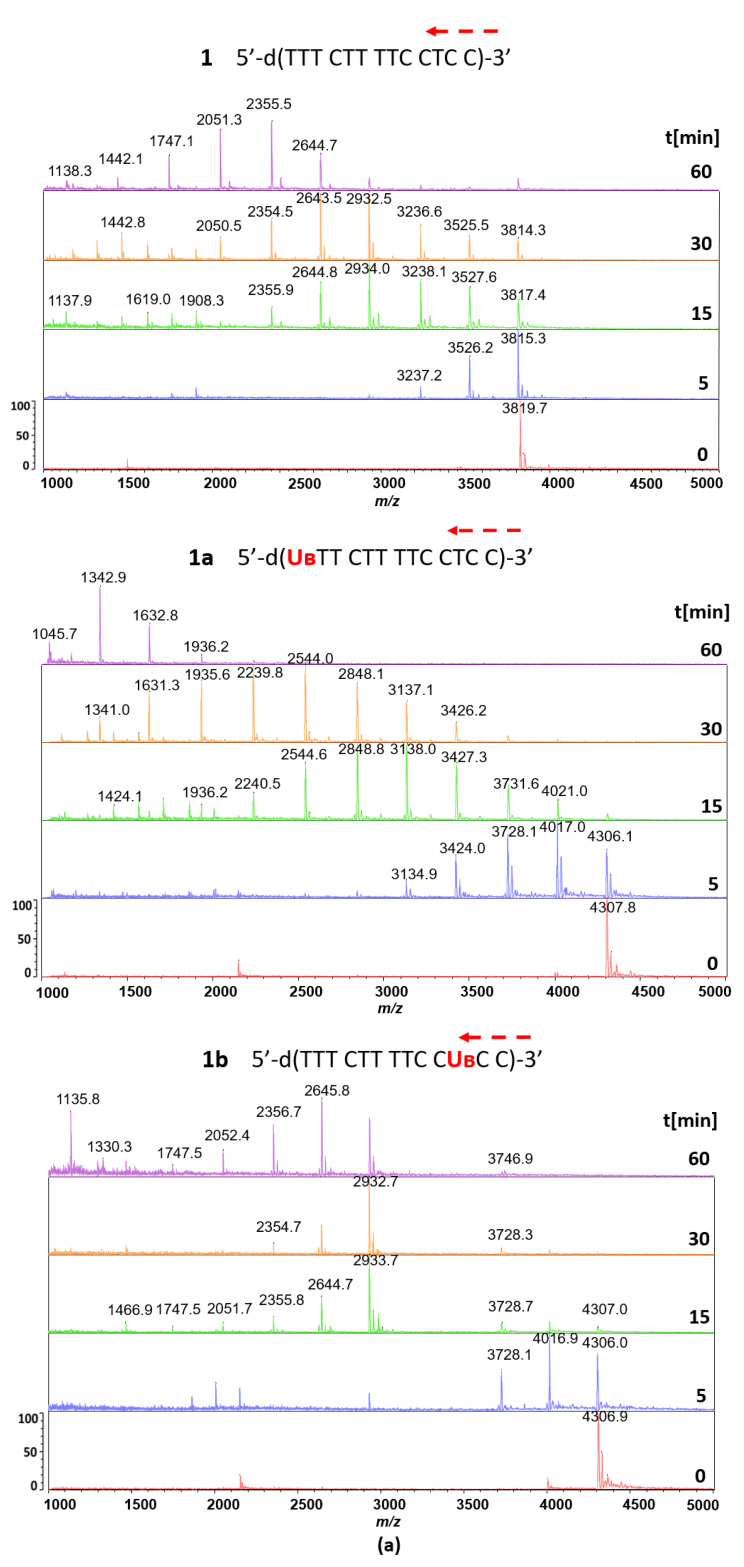

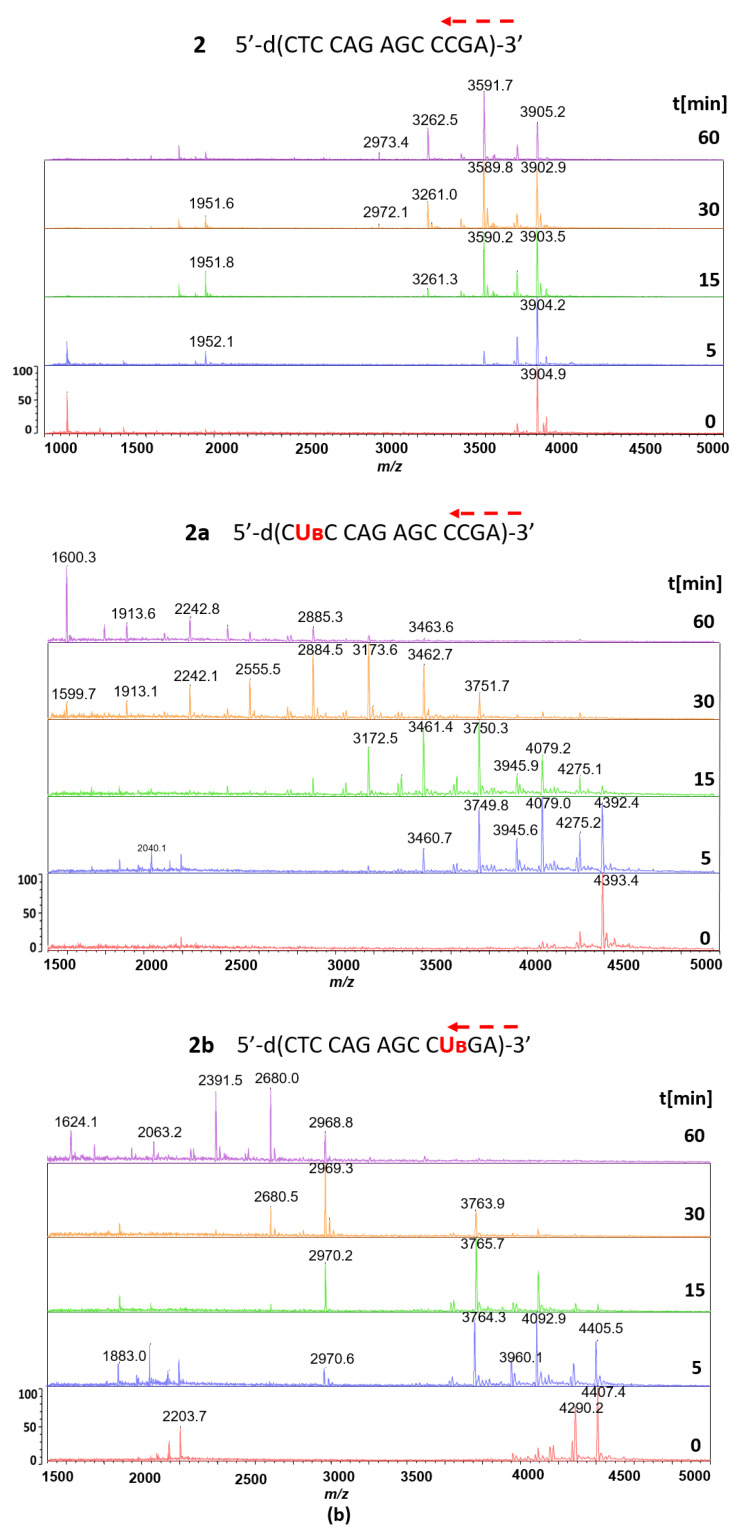
MALDI-TOF mass spectra of the hydrolysates of oligonucleotides **1**, **1a, 1b** (panel **a**) **2**, **2a** and **2b** (panel **b**) after 0, 5, 15, 30 and 60 min of treatment with svPDE. The experimental conditions are described in the Materials and Methods section.

In addition, ^32^P-phosphate-free **1**, **1a**, **1b**, **2**, **2a** and **2b** were treated with svPDE, and the hydrolysates collected at 0, 5, 15, 30 and 60 min time points were analyzed by MALDI-TOF mass spectrometry (Figure 3). The results are well correlated with those obtained from PAGE and indicate that the metallacarborane complex enhanced the rate of cleavage by svPDE (at least until the modified site was cleaved off) because B-ASOs are degraded faster than their parent oligomers **1** and **2**.

**Figure 4:**
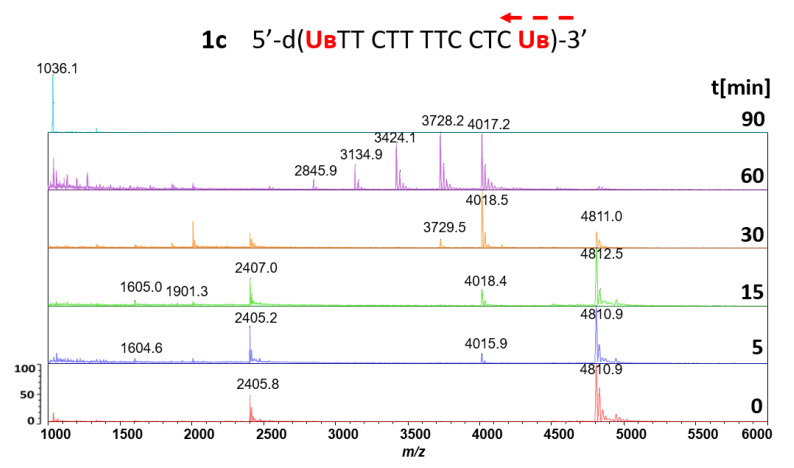
MALDI-TOF mass spectra of the hydrolysates of oligonucleotide **1c** after 0, 5, 15, 30, 60 and 90 min of treatment with svPDE. The experimental conditions are described in the Materials and Methods section.

Furthermore, we also checked the svPDE-assisted hydrolytic stability of oligonucleotide **1c** containing two metallacarborane units at the ultimate 5’- and 3’-terminal positions (1 and 13 from the 5’-end, respectively). As shown in Figure 4, the **U_B_** unit at the 3’-end was effectively cleaved by svPDE, with slightly slower kinetics than that for **1**. At 30 min, ca. 50% of **1c** remained in the hydrolysis mixture (T_1/2_ ca. 30 min versus T_1/2_ 14.5 min for **1**) (Figure S4). Interestingly, the remaining shorter product with *m/z* 4017.2 was cleaved in the next 30 min to afford dimer 5’-**U_B_**T-3’ with *m/z* 1036.1, further confirming the stimulatory activity of the metallacarborane unit located at the 5’-end of B-ASO.

### 3.4. svPDE-Catalyzed Cleavage of an Equimolar Mixture of ***1*** + ***1a***

**Figure 5:**
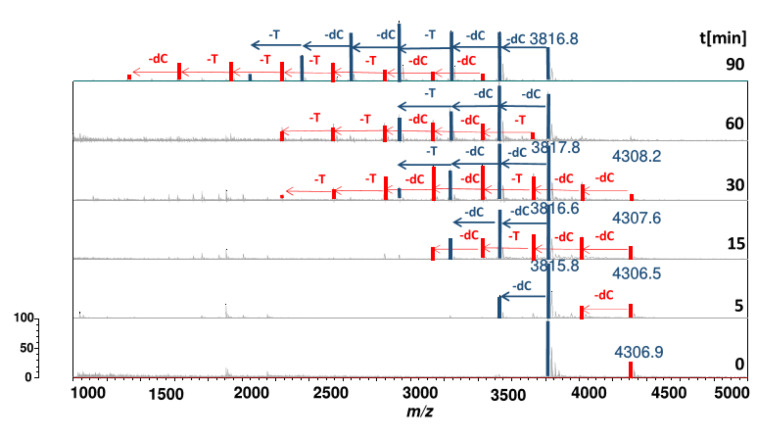
MALDI-TOF MS spectra of the hydrolysates obtained after the treatment of the equimolar mixture of oligonucleotides **1** (blue peaks) and **1a** (red peaks) with svPDE. Spectra of aliquots taken at 0, 5, 15, 30, 60 and 90 min were acquired.

To obtain more quantitative data on the cleavage rates of nonmodified and modified oligomers, we analyzed the cleavage patterns of **1** and **1a** in a time-course experiment in which both oligomers (mixed at a 1:1 molar ratio) were degraded by svPDE. Interestingly, MALDI-TOF MS analysis at time point 0 revealed that relative intensity of the peaks which corresponds to [M-H]^-^ ions were far from equal, as a 3:1 ratio was noted (see Figure 5 at time 0 min, and Figures S5 and S6). This indicates that the metallacarborane unit not only slows the migration of the labeled oligonucleotides in the polyacrylamide gel (vide supra) but also substantially reduces their vaporization from the matrix used in the reported MALDI-TOF MS experiments. This observation was important for the interpretation of the spectra recorded after 5, 15, 30 and 60 min. A comparison of the intensities of the peaks at particular time points (Figure 5) clearly indicates that the band for **1a** (at *m/z* 4306.9) disappears much faster than that for **1** (at *m/z* 3816.8), and at 60 min, the former is barely visible, whereas the latter is almost the most intense peak in the mass spectrum.

**Figure 6:**
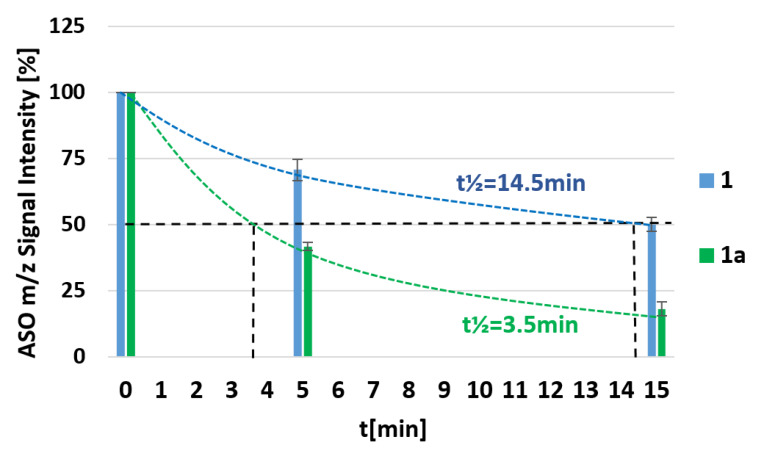
Analysis of the hydrolysis rates of **1** and **1a** (1:1 mixture) in the presence of svPDE. The % contents of intact **1** and **1a** were calculated after 0, 5 and 15 min of hydrolysis based on the relative intensities of the peaks in MALDI-TOF MS spectra. The sums of the relative intensity of the peaks corresponds to the intact oligomers and their two major fragments (12 nt and 11 nt) were taken as 100% at each time point. SD ± values are marked. The roughly estimated T_1/2_ values were assessed, as shown.

Although mass spectrometry is not suitable for quantitative analysis over large *m/z* ranges, some valuable data can be obtained by comparing peaks for products with similar molecular masses, provided that the chemical character of the ions being compared is strictly retained. Thus, from the spectra recorded at 0, 5 and 15 min of hydrolysis (Figure 5), we calculated the percentages of the bands for intact **1** and **1a** based on the sums of the relative intensities of their bands and those measured for their two major fragments (12 nt and 11 nt). The resultant plot (Figure 6; data obtained from three independent repetitions) showed that **1a** was cleaved ca. 4 times faster than nonmodified ASO **1**, with roughly estimated T_1/2_ = ca 3.5 and 14.5 min, respectively.

### 3.5. Enhanced 3’→5’ Exonucleolytic Activity of svPDE in the Presence of Free Metallacarborane

**Figure 7:**
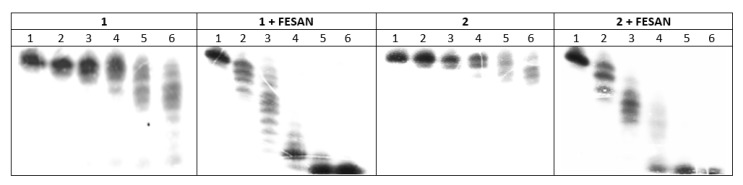
Autoradiograms after denaturing PAGE, showing the products of svPDE-promoted hydrolysis of ^32^P-labeled oligonucleotides **1** or **2** mixed with metallacarborane ferra(III) bis(dicarbollide) (6 molar equivalents relative to ASO). Lanes 1 to 6 represent the products present in aliquots of the hydrolysis reaction taken at 0, 5, 15, 30, 60 and 90 min, respectively.

Having observed the enhanced activity of svPDE, we decided to check whether the metallacarborane itself may similarly accelerate the cleavage reaction catalyzed by the enzyme. To this end, ^32^P-phosphate-labeled oligonucleotides **1** and **2** (0.10 OD; 0.91 nmol and 0.75 nmol, respectively), were mixed with 6 molar equivalents of ferra(III) bis(dicarbollide) (FESAN) and incubated with svPDE (1 μL, 0.5 mU). The PAGE experiments (the relevant autoradiograms are shown in Figure 7) revealed that after 30 min of incubation with the metallacarborane, both substrates were degraded completely, whereas those hydrolyzed without the boron species were truncated by less than 6 nucleotides. These results were confirmed by MALDI TOF-MS analysis (Figure S7).

Next, we checked whether the observed phenomenon is dependent on the metallacarborane concentration by testing the cleavage rate of **1** in the presence of svPDE (both at constant concentrations) and increasing concentrations of ferra(III) bis(dicarbollide). Here, we used a mass spectrometry technique and analyzed in triplicate the profiles of the cleavage products at 0, 5 and 15 min for the reactions with 1-, 2- and 3-fold molar excess of metallacarborane over **1** (Figure S8).

**Figure 8:**
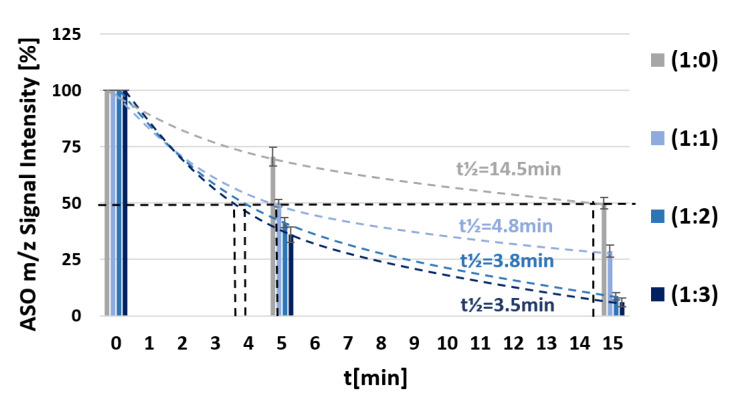
Analysis of the hydrolysis rate of oligonucleotide **1** (1 equiv.) in the svPDE-assisted reaction carried out in the presence of 0, 1, 2 or 3 equivalents of metallacarborane. The % content of intact **1** was calculated after 0, 5 and 15 min of hydrolysis based on the relative peak intensities in the MALDI-TOF MS spectra. The sums of the relative intensity of the peaks corresponds to the intact oligomers and their two major fragments (12 nt and 11 nt) were taken as 100% at each time point. SD± values are marked. The roughly estimated T_1/2_ values were assessed, as shown.

The experiments demonstrated that, indeed, the rate of the hydrolysis reaction is dependent on the metallacarborane concentration. The hydrolysis rate increased gradually as the excess of metallacarborane increased up to 2-fold. With 3 equivalents, the rate of the reaction increased only minimally, which suggests that the reaction system becomes saturated. The half-lives (T_1/2_ roughly estimated values) of the hydrolysis rate dropped from ca. 14.5 min to ca. 4.8 min, 3.8 min and 3.5 min for 1:0 to 1:1, 1:2 and 1:3 molar ratios of **1** to metallacarborane complex, respectively (Figure 8).

A summary of the half-lives (T_1/2_) of all oligonucleotides tested is given in Table 2.

### 3.6. Affinity of Ferra(III) bis(dicarbollide) for Proteins Present in Crude Snake Venom Used as a Source of svPDE

**Figure 9:**
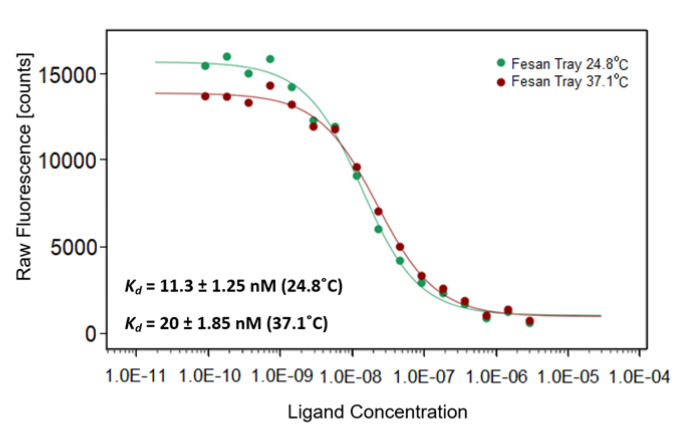
Curves obtained from microscale thermophoresis experiments aimed assessing the strength of the interactions between ferra(III) bis(dicarbollide) and the venom proteins fluorescently labeled with RED-NHS 2nd generation. The protein samples were titrated with ferra(III) bis(dicarbollide) at concentrations ranging from 182 pM to 3000 nM. The experiments were run at 24.8 °C and 37.1 °C. The ligand concentration is given in M.

This work used crude *Crotalus atrox* venom, which is a mixture of several enzymes, including phosphomono- and diesterases, acetylcholinesterases, NAD-nucleosidases, endonucleases, metallo- and serine-proteinases, as well as nonenzymatic proteins such as VEGF, C-type lectins, disintegrins, myotoxins, and protease inhibitors [32]. The content of svPDE in crude venom is very low [33]; we found it to be only approximately 0.018% (see PAGE analysis in Figure S9 for the quantification of the content of svPDE with MW 112 kDa). Despite this low amount of pure svPDE, we decided to determine the binding affinity of ferra(III) bis(dicarbollide) to the total venom proteins using microscale thermophoresis. The mixture of proteins at 0.5 mU of total svPDE concentration was treated with a RED-NHS 2nd generation fluorescent dye. The dye carries a reactive NHS-ester group that forms a covalent bond with primary amines (lysine residues). According to a recently published biocomputational work [34], the surface of svPDE from *Crotalus Atrox*, which is a close analog of the svPDE used in this work, has many lysine residues, so their labeling should be quite effective. In the subsequent MST experiment, ferra(III) bis(dicarbollide) at concentrations of 182 pM to 3000 nM was used as the titrant. The experiments were run at 24.8 °C and 37.1 °C, and the obtained data points gave rise to curves of satisfactory quality (Figure 9). The detailed experimental data, including capillary scans, are given in the Supplementary Material (Figure S10). The calculated dissociation constants (*K_d_*) of 11.3 ± 1.25 nM (green curve, data collected at 24.8 °C) and 20 ± 1.85 nM (red curve, at 37.1 °C) indicate strong affinities between ferra(III) bis(dicarbollide) and the venom proteins.

### 3.7. Affinity of Ferra(III) bis(dicarbollide) to 5’-Fluorescently Labeled Oligonucleotides ***1*** and ***2***

**Figure 10:**
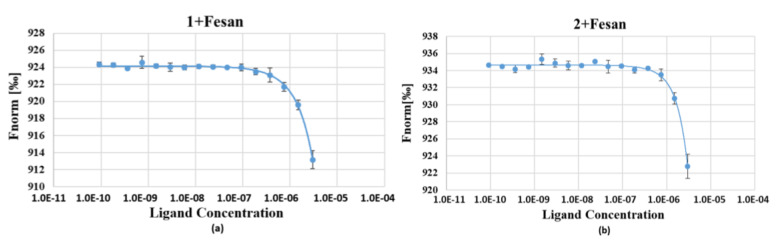
Curves obtained from microscale thermophoresis experiments aimed at assessing the affinity of ferra(III) bis(dicarbollide) (FESAN) for fluorescently labeled oligonucleotides **FL-1** (**a**) or **FL-2** (**b**). The oligonucleotide samples were titrated with ferra(III) bis(dicarbollide) at concentrations ranging from 182 pM to 3000 nM. The experiments were run at 22 °C. The ligand concentration is given in M.

The affinities of ferra(III) bis(dicarbollide) to short single-stranded DNA oligonucleotides **1** and **2** labeled with fluorescent tags inserted at their 5’-ends (compounds **FL-1** and **FL-2**) were analyzed by MST (Figure 10a, b respectively). Here, the titration of the fluorescent probes of the oligonucleotides with ferra(III) bis(dicarbollide) required much higher concentrations of ligand (above 6 µM) compared to the experiment described above, and at these concentrations, the tested ferra(III) bis(dicarbollide) started to precipitate. Thus, these conditions did not allow us to obtain curves with clear MST transitions that can be used to calculate exact *K_d_* values. However, metallacarborane clearly exhibits reasonable affinity to this single-stranded DNA. Extrapolation of the obtained curves allowed us to determine an approximate *K*_d_ = 5.48 ± 0.20 µM value for oligonucleotide **FL-1** (see Figure S11). Importantly, the profiles of the MST curves for both fluorescent oligomers are similar, which may indicate that the affinity of metallacarborane to DNA is independent of its sequence.

## 4. Discussion

To date, most of the information available on the biological activities of metallacarboranes (mostly cobalt bis(1,2-dicarbollide) (COSAN)) is related to possible medical applications. These complexes may serve as boron sources for BNCT [35,36]. In addition, boron clusters and their conjugates with nucleosides exhibit high binding affinity for plasma proteins under in vitro and in vivo conditions [37,38] as well as the ability to cross bilayer membranes and to interact with cell membranes [39]. Recently, it was shown that a conjugate of cobalt bis(1,2-dicarbollide) and a Tβ4 protein (thymosin β4) incubated with cardiomyocytes increased the viability of hypoxic rat heart cells [40] and enhanced the wound healing potential of Tβ4 in human fibroblasts [41]. Also, our studies demonstrated that the presence of metallacarborane (FESAN) either linked to ASO or to nucleoside moiety increased mitochondrial activity of the cells up to ~20%–40% (MTT test) [19,20]. The free metallacarborane exhibited similar potency for enhancement of mitochondrial activity (up to 50%) [20], but caused an activation of inflammasome (IL-1 β) in macrophages differentiated from monocytes isolated from human blood of healthy donors (up to 40%, ELISA test, data not shown).

Over the last several years, we investigated the effects of the attachment of boron clusters to antisense oligonucleotides rather than to nucleosides, exploring the idea that in anticancer therapies, the inhibition of gene expression may act synergistically with the BNCT phenomenon. The goal of the present work was to verify the assumption that metallacarborane-modified oligonucleotides may be less prone to nucleolytic degradation than native oligomers, as was observed in the past for oligonucleotides modified with other boron clusters [22,24,27]. This phenomenon was expected to be more pronounced for oligomers with metallacarboranes located closer to their 3’-ends. Because the initial results of the hydrolysis of 22-nt oligonucleotides (ASO-22) containing different boron clusters were unclear (data not shown), we decided to use shorter models **1a**-**1c** and **2a**, **2b** derived from polypyrimidine **1** and purine-enriched **2**, respectively, by the addition of one or two ferra(III) bis(dicarbollide) moieties. The cage was attached to the uridine units by “click” chemistry, with the formation of a 10-atom-long linker. As a model 3’→5’-exonuclease, snake venom phosphodiesterase (svPDE, an enzyme with 3’→5’-exonucleolytic activity) routinely used in such studies was chosen.

Unexpectedly, it was found that upon treatment with svPDE, the ferra(III) bis(dicarbollide)-labeled oligomers (**1a** and **1b** as well as **2a** and **2b**), independent of the cage location (either close to the 5’-end as in **1a** and **2a**, or close to the 3’-terminus, as in **1b** and **2b**), were degraded faster than parent compounds **1** and **2** (see Figure 1, Figure 2 and Figure 3). This was well documented in the experiment in which a 1:1 molar mixture of **1** and **1a** was hydrolyzed, and the reaction progress was monitored by MALDI-TOF MS (Figure 5 and Figure 6). It was also found that from the moment the units bearing the cage (**U_B_**) in **1b** and **2b** were removed, the remaining fragments were hydrolyzed slower, at a rate similar to that of the nonmodified oligomers. Interestingly, the relatively bulky **U_B_** unit at the 3’-ASO neither prevented cleavage at the modification site (see data for **1b**, **1c** and **2b**), as was demonstrated for other boron clusters [29], nor produced the p**U_B_**pN dinucleotide that was otherwise expected from the known hopping property of the enzyme [42]. It is worth noting that in the case of other boron cluster modifications, they were located close to the cleaved internucleotide bond: either at the 5-uracil position, 2’-ribose, or even on a phosphate group. In this work, the metallacarborane was always attached by a fairly long linker; hence, its “bulkiness” may not matter.

The phenomenon observed here seems to be related to the aforementioned ability of carborane cages to bind (reversibly) with proteins [35,37]. In addition, the interactions between cobalt bis(1,2-dicarbollide) and dsDNA (from calf thymus) was univocally demonstrated, as shown by IR, Raman, and UV spectroscopic studies [38]. The melting curves confirmed this interaction as being dependent on the ionic strength of the solution. Of course, one cannot exclude that in our experiments, the cages might produce an allosteric effect, so one molecule of labeled oligonucleotide would provide the cage to generate an allosteric form of the enzyme and the other molecules would then be hydrolyzed faster; however, this explanation should be rejected in light of the result of the hydrolysis of the **1**/**1a** mixture. Additionally, such “accidental upgrading” of the evolutionarily highly developed protein is close to impossible. This option was further dismissed based on experiments where the free Fe^3+^-containing bis(dicarbollide) complex (FESAN), which was used in excess relative to the oligonucleotide substrates, enhanced the svPDE-promoted hydrolysis of **1** and **2** (Figure 7, Figure 8 and Figure S8). Keeping in mind the abovementioned facts, we concluded that the metallacarborane cage acts as an interface (the “glue”) between a single-stranded DNA and the protein molecule. If the cage is covalently linked with the DNA oligomer, the reversible association/dissociation process holds the substrate close to the enzyme, giving rise to a higher local concentration of substrate near the enzyme compared to that of unlabeled oligomers. Nonetheless, if the association constant is not very high, they retain an ability to “slide” over the oligopeptide surface and, eventually, to enter the catalytic pocket. If the free ferra(III) bis(dicarbollide) is delivered, its molecules associate with the protein and in this way form a “connector” that can attract DNA molecules.

The relatively high affinity of negatively charged ferra(III) bis(dicarbollide) for crude snake venom proteins has been confirmed by MST measurements (Figure 9). We also confirmed the binding of ferra(III) bis(dicarbollide) to oligonucleotides **1** and **2**, although in this case, the affinity constant was approximately three orders of magnitude lower than that for the proteins (Figure 10). Because of the excess boron cages, the protein surface may be covered to an extent [43] sufficient for facilitating the “sliding” of DNA into the pocket. Although the boron-cluster saturation effect of the crude svPDE was observed for a 3-fold molar excess of metallacarborane relative to hydrolyzed oligonucleotide (Figure 8), both processes would lead to the same increase in the local concentration of DNA, which is macroscopically indicated by the acceleration of the hydrolysis. In this context, the affinity of the negatively charged metallacarborane for proteins may explain the acceleration of the hydrolysis observed in these studies and the inhibition of the svPDE catalytic activity by other cages, as observed previously [24,44].

Our discovery for the first time demonstrates that both metallacarboranes conjugated with nucleic acid fragments and free metallacarborane cages, tested in the system described here, increase the rate of reactions catalyzed by phosphodiesterase, which belongs to the class of enzymes involved in cell metabolism and interacting with nucleic acid components. This result is directly related to the medical applications of ASO oligonucleotides, which might be protected by previously established approaches, for example, by PS-internucleotide bond modifications, while boron cluster pedants might keep the reactant biomolecules in proximity to enhance the affinity of nucleic acid fragments for the interacting protein partners, facilitating therapeutic effects. Our preliminary observations showed that in some cases, target RNA chains in RNA/DNA heteroduplexes bearing carborane clusters in the antisense part were digested more efficiently by RNase H than were their unmodified counterparts [24,44]. Moreover, in the cellular experiments B-ASOs bearing metallacarborane were more active anti-EGFR inhibitors comparing unmodified ASO and B-ASOs containing 1,2-dicarba-*closo*-dodecaborane (C_2_B_10_H_12_) [19]. One can envisage that antisense oligomers modified with metallacarboranes may assist RNase H cleavage more efficiently than carborane-containing oligonucleotides due to their higher affinity towards proteins than carboranes [37].

Currently, our laboratory is trying to exploit the discovered phenomenon and use boron clusters and their conjugates to increase the catalytic efficiency of selected enzymes and cellular processes.

## 5. Conclusions

In this work, we advanced the scientific understanding of the properties of metallacarborane clusters by demonstrating their ability to enhance the rate of the hydrolysis of DNA oligonucleotides catalyzed by snake venom phosphodiesterase. This phenomenon was demonstrated on single-stranded DNA oligonucleotides either conjugated with ferra(III) bis(dicarbollide) or incubated with a free metallacarborane. The rate of the hydrolysis reaction was dependent on the concentration of ferra(III) bis(dicarbollide), and magnesium ions were required for the catalytic activity of the svPDE enzyme. The affinity of ferra(III) bis(dicarbollide) towards proteins, as demonstrated previously in the literature, and confirmed by us on a crude venom protein mixture as well as towards DNA, and confirmed here on short DNA oligonucleotides, offers an explanation for its participation in attracting both reactant biomolecules, accelerating the rate of the reaction undergoing between the DNA and protein partners (svPDE). If so, this discovery is of paramount importance for applications of boron clusters in processes in which oligonucleotides and cellular proteins are required to achieve biological effects, e.g., enhancing the activity of therapeutic nucleic acids. The studies on the effect of metallacarborane and boron clusters on other cellular processes and enzymes interacting with nucleic acids are in progress in our Laboratory.

## Figures and Tables

**Table 1:** Sequences, molecular weight, and chromatographic mobility (RP-HPLC) data for the oligonucleotide substrates used in these studies. **U_Pr_** and **U_B_** represent 2’-*O*-propargyl uridine and metallacarborane-containing uridine units, respectively.

No.	Sequences	MW Calc. (g/mol)	MALDI-TOF MS (*m/z*)	RP-HPLC (Rt, min)
**1**	5’-d(TTT CTT TTC CTC C)-3’	3817.50	3817.98 ^1^	13.04
**2**	5’-d(CTC CAG AGC CCG A)-3’	3904.56	3904.82 ^1^	12.27
**P1a**	5’-d(U_Pr_TT CTT TTC CTC C)-3’	3857.52	3857.98 ^1^	13.13
**P1b**	5’-d(TTT CTT TTC CU_Pr_C C)-3’	3857.52	3858.02 ^1^	13.14
**P1c**	5’-d(U_Pr_TT CTT TTC CTC U_Pr_)-3’	3912.55	3910.80	13.51
**P2a**	5’-d(CU_Pr_C CAG AGC CCGA)-3’	3944.58	3945.20	12.06
**P2b**	5’-d(CTC CAG AGC CU_Pr_GA)-3’	3959.59	3958.40	12.15
**1a**	5’-d(U_B_ TT CTT TTC CTC C)-3’	4307.29	4306.20	25.20
**1b**	5’-d(TTT CTT TTC C U_B_C C)-3’	4307.29	4305.90	23.67
**1c**	5’-d(U_B_TT CTT TTC CTCU_B_)-3’	4812.11	4810.90	29.12
**2a**	5’-d(CU_B_C CAG AGC CCGA)-3’	4394.36	4393.70	24.39
**2b**	5’-d(CTC CAG AGC CU_B_GA)-3’	4409.37	4411.10 ^1^	23.95
**FL-1**	5’-FL-d(TTT CTT TTC CTC C)-3’	4354.95	4355.70 ^1^	15.57
**FL-2**	5’-FL-d(CTC CAG AGC CCG A)-3’	4442.02	4442.65 ^1^	14.54

^1^ Analysis was performed by electrospray ionization mass spectrometry (ESI MS). The raw ESI mass spectra were deconvoluted using the MaxEnt1 algorithm to a zero-charge state mass.

**Table 2:** Nucleolytic stability of oligonucleotides tested in these studies. The values of the half-lives (T_1/2_) are given for the hydrolysis reactions carried out in the presence of svPDE.

ASO	Sequence 5’ → 3’	T_1/2_ (min)
**1**	5’-d(TTT CTT TTC CTC C)-3’	14.5
**2**	5’-d(CTC CAG AGC CCG A)-3’	25.0
**1a**	5’-d(U_B_ TT CTT TTC CTC C)-3’	3.5
**1b**	5’-d(TTT CTT TTC C U_B_C C)-3’	4.0
**1c**	5’-d(U_B_TT CTT TTC CTCU_B_)-3’	20.0
**2a**	5’-d(CU_B_C CAG AGC CCGA)-3’	3.5
**2b**	5’-d(CTC CAG AGC CU_B_GA)-3’	4.0
**1**	5’-d(TTT CTT TTC CTC C)-3’ + FESAN (1 equiv.)	4.8
**1**	5’-d(TTT CTT TTC CTC C)-3’ + FESAN (2 equiv.)	3.8
**1**	5’-d(TTT CTT TTC CTC C)-3’ + FESAN (3 equiv.)	3.5

## Supplementary Materials

The following are available online at https://www.mdpi.com/2218-273X/10/5/718/s1. Figure S1: Analytical RP HPLC profiles and MALDI-TOF or ESI-Q-TOF mass spectra of **1**, **P1a**, **P1b**, **P1c**, **1a**, **1b**, **1c**, **2**, **P2a**, **P2b**, **2a**, **2b**, **FL-1** and **FL-2**. Figure S2: PAGE analysis of the **1**, **1a**, **1b**, **1c** and **2**, **2a**, **2b** oligonucleotides (20%/7 M urea). Figure S3: svPDE-assisted hydrolysis of the reference oligonucleotide 1 with and without magnesium ions, monitored by MALDI-TOF mass spectrometry. Figure S4: Original MALDI-TOF MS spectra of hydrolysis of **1c** in the presence of svPDE (in triplicate). Figure S5: MALDI-TOF mass spectrometry analysis of the 1:1:1 mixture of **1**, **1a** and oligonucleotide **1** containing two FESAN clusters. Figure S6: Original MALDI-TOF MS spectra of hydrolysis of **1**+**1a** in the presence of svPDE (in triplicate). Figure S7: MALDI-TOF MS analysis of reaction mixture of the svPDE-assisted hydrolysis of unmodified oligonucleotides **1** and **2** in the presence of a free metallacarborane. Figure S8: MALDI-TOF analysis of the hydrolysis rate of oligonucleotide **1** in the svPDE-assisted reaction carried out in the presence of 0, 1, 2 or 3 equivalents of metallacarborane (in triplicate). Figure S9: PAGE analysis of the crude snake venom. Figure S10: The protein samples capillary scan from microscale thermophoresis experiments (the venom proteins fluorescently labeled with RED-NHS 2nd generation). Figure S11: MST data for *K*d determination of affinity of FESAN (ferra(III) bis(dicarbollide)) to **FL-1**.
