# Supplementary material for: Metallacarborane Complex Boosts the Rate of DNA Oligonucleotide Hydrolysis in the Reaction Catalyzed by Snake Venom Phosphodiesterase
Source: Biomolecules. 2020 May 5;10(5):718. doi: 10.3390/biom10050718 (PMC7277537; doi:10.3390/biom10050718)
Supplement: Supplementary file 1 [file biomolecules-10-00718-s001.pdf]

Supplementary Material

Metallacarborane complex boosts the rate of DNA oligonucleotide hydrolysis in the reaction catalyzed by snake venom phosphodiesterase

Damian Kaniowski[1], Katarzyna Kulik[1], Katarzyna Ebenryter-Olbińska[1], Ewelina Wielgus[1], Zbigniew Leśnikowski[2], Barbara Nawrot [1,*]

[1]Centre of Molecular and Macromolecular Studies, Polish Academy of Sciences, Sienkiewicza 112, 90-363 Lodz, Poland
[2]Institute of Medical Biology, Polish Academy of Sciences, Laboratory of Medicinal Chemistry, 106 Lodowa St., 92-232 Lodz, Poland

Figure S1. Analytical RP HPLC profiles and MALDI-TOF or ESI-Q-TOF (*) mass spectra of **1**, **P1a**, **P1b**, **P1c**, **1a**, **1b**, **1c**, **2**, **P2a**, **P2b**, **2a, 2b, FL-1** and **FL-2.**
RP-HPLC conditions were as follow: the buffer A (0.1 M CH_3COONH_4) and buffer B (100% CH_3CN). The buffer B gradient: 0→2 min 0%; 2→25 min 0-48%; 25→30 min 48-60%; 30→35 min 60-0%; 35→38 min 0%.

Figure S2. PAGE analysis of the **1, 1a, 1b, 1c** and **2, 2a, 2b** oligonucleotides (20%/7 M urea)

Figure S3. svPDE - assisted hydrolysis of reference oligonucleotide **1** with and without magnesium ions, monitored by MALDI-TOF mass spectrometry.

Figure S4. Original MALDI-TOF MS spectra of hydrolysis of **1c** in the presence of svPDE (in triplicate).

Figure S5. MALDI-TOF mass spectrometry analysis of the 1:1:1 mixture of **1, 1a** and oligonucleotide **1** containing two FESAN clusters (synthetic data not shown). Intensity of peaks is given on the Figure.

Figure S6. Original MALDI-TOF MS spectra of the hydrolysis mixture of **1**+**1a** in the presence of svPDE (in triplicate).

Figure S7. Figure S7. MALDI-TOF MS analysis of the reaction mixture of svPDE-assisted hydrolysis of unmodified oligonucleotides 1 and 2 (0.1 OD) in the presence of a free metallacarborane (FESAN, 182 nM) at 0-60 min.

Figure S8. MALDI-TOF MS analysis of the hydrolysis rate of oligonucleotide **1** in the svPDE-assisted reaction carried out in the presence of 0, 1, 2 or 3 equivalents of metallacarborane (in triplicate)
Figure S9. PAGE analysis of crude snake venom.
Figure S10. The protein samples capillary scan from microscale thermophoresis (the venom proteins fluorescently labeled with RED-NHS 2nd generation).
Figure S11. MST data for K_d determination of affinity of FESAN (ferra(III) bis(dicarbollide)) to **FL-1** (K_d=5.48±0.20 µM).

Figure S1. Analytical RP HPLC profiles and MALDI-TOF or ESI-Q-TOF (*) mass spectra of **1**, **P1a**, **P1b**, **P1c**, **1a**, **1b**, **1c**, **2**, **P2a**, **P2b**, **2a, 2b, FL-1** and **FL-2.**

RP-HPLC conditions were as follow: the buffer A (0.1 M CH_3COONH_4) and buffer B (100% CH_3CN). The buffer B gradient: 0→2 min 0%; 2→25 min 0-48%; 25→30 min 48-60%; 30→35 min 60-0%; 35→38 min 0%.

1 5’-d(TTT CTT TTC CTC C)-3’ (*)

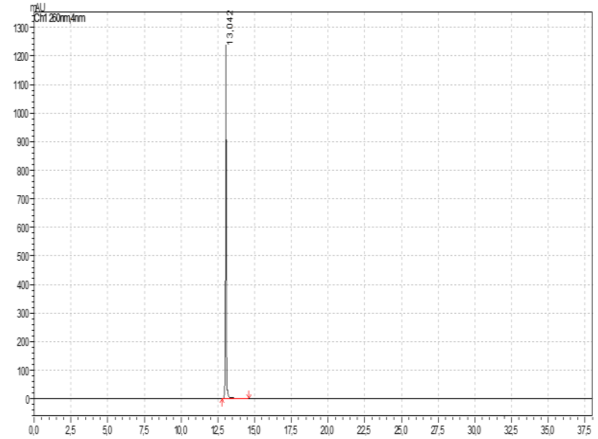

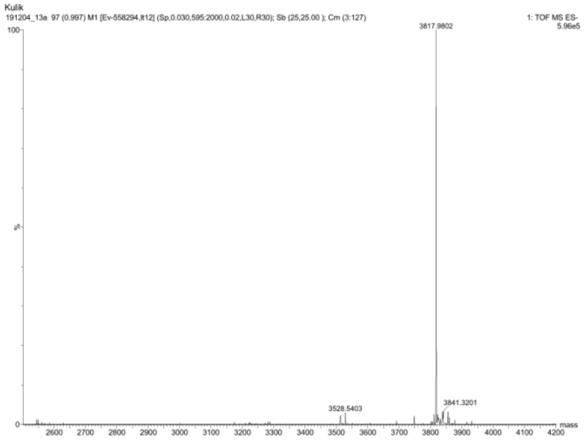

P1a 5’-d($\mathbf{U_{Pr}}$TT CTT TTC CTC C)-3’ (*)

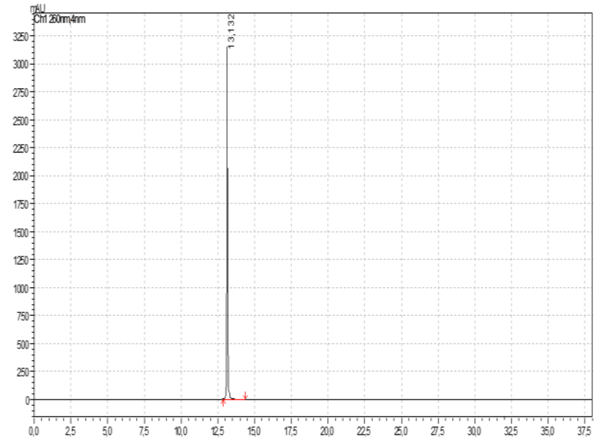

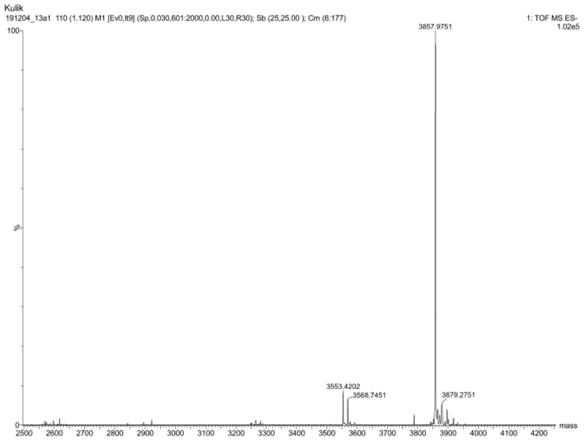

1a 5’-d(U_BTT CTT TTC CTC C)-3’

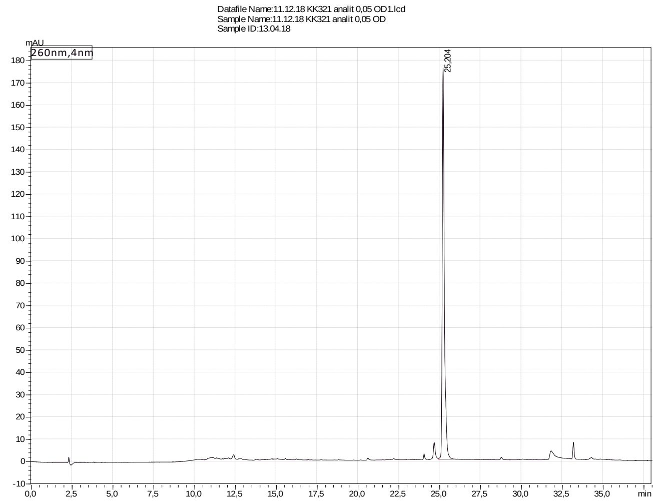

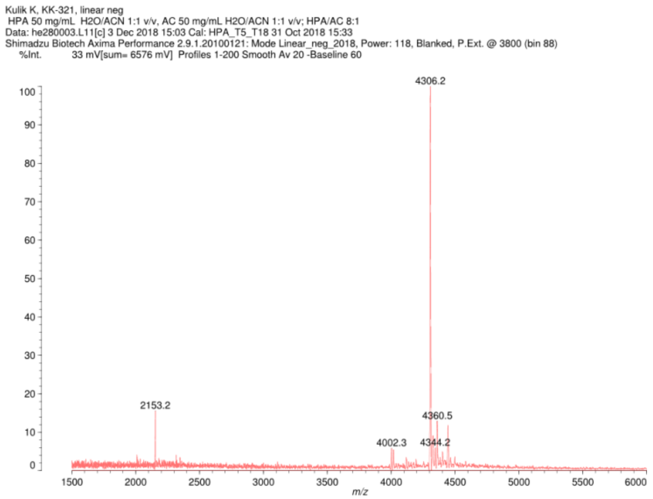

P1b 5’-d(TTT CTT TTC CU_{Pr}C C)-3’

(*)

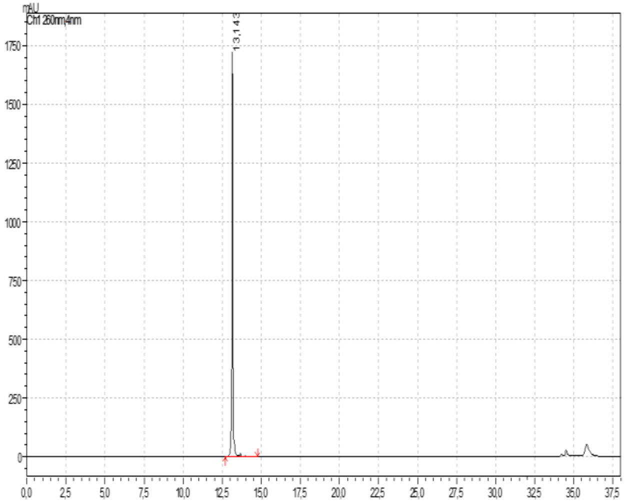

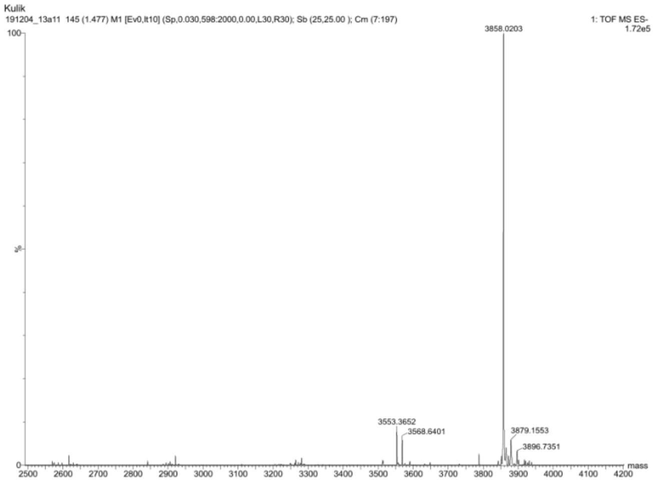

1b 5'-d(TTT CTT TTC C**U**$_{\mathbf{B}}$C C)-3'

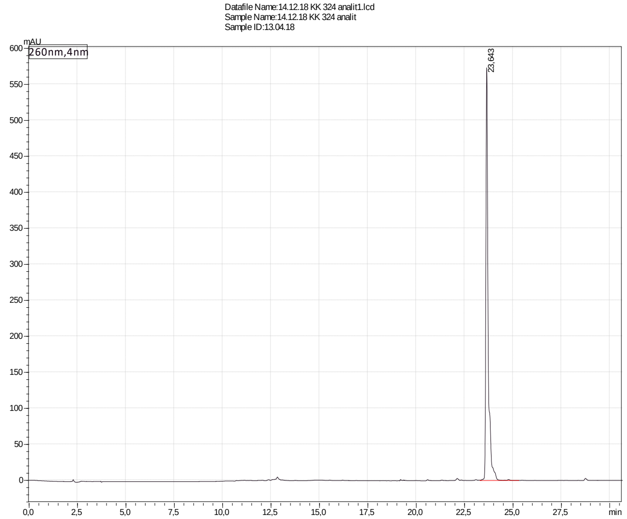

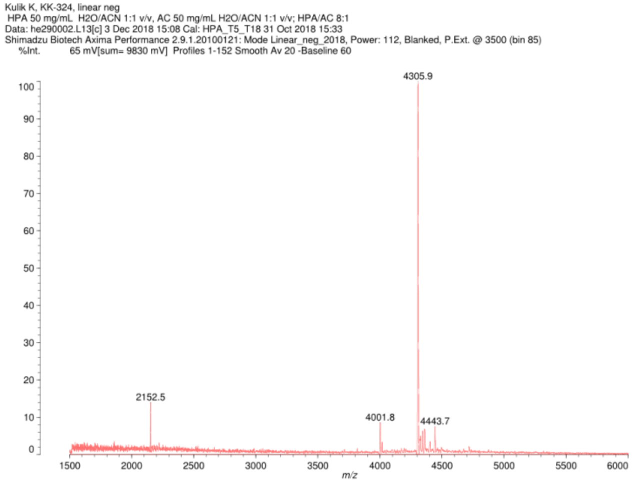

P1c 5'-d(**U**$_{\mathbf{Pr}}$TT CTT TTC CTC **U**$_{\mathbf{Pr}}$)-3'

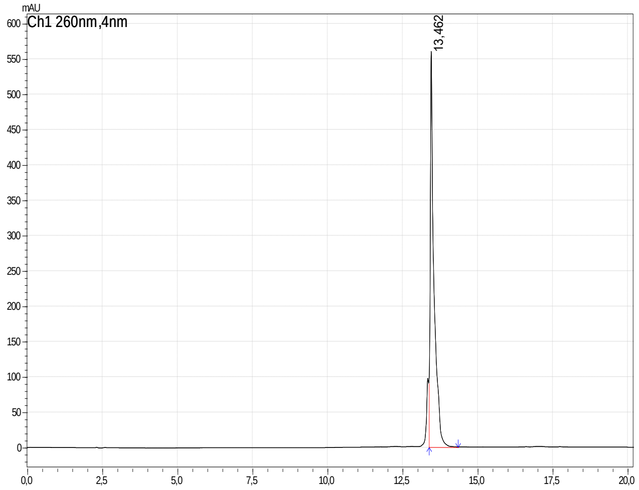

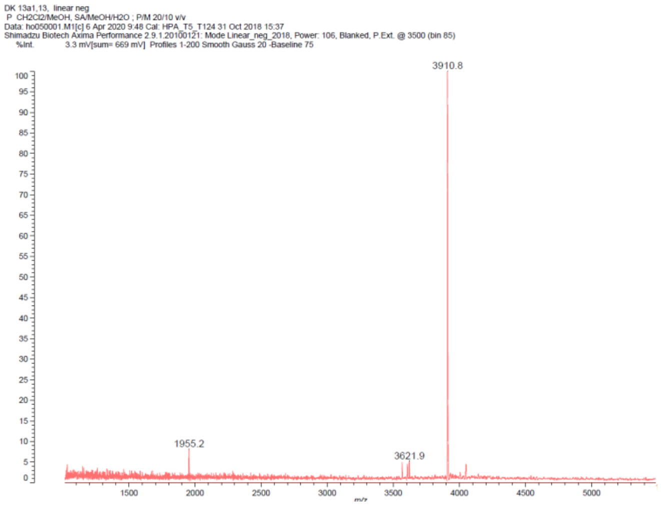

1c 5'-d(**U**$_B$TT CTT TTC CTC**U**$_B$)-3'

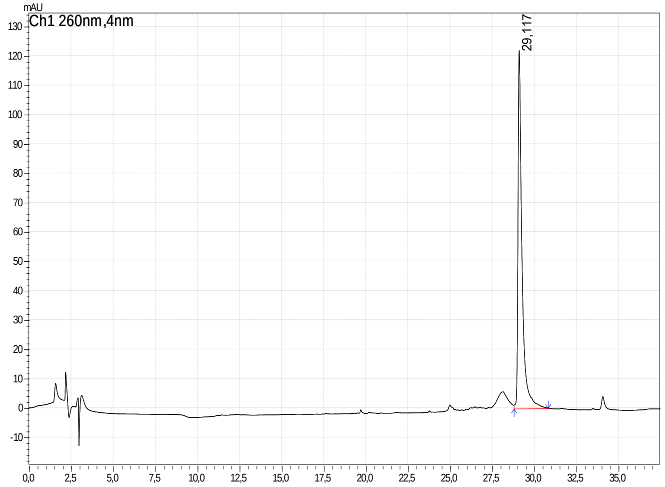

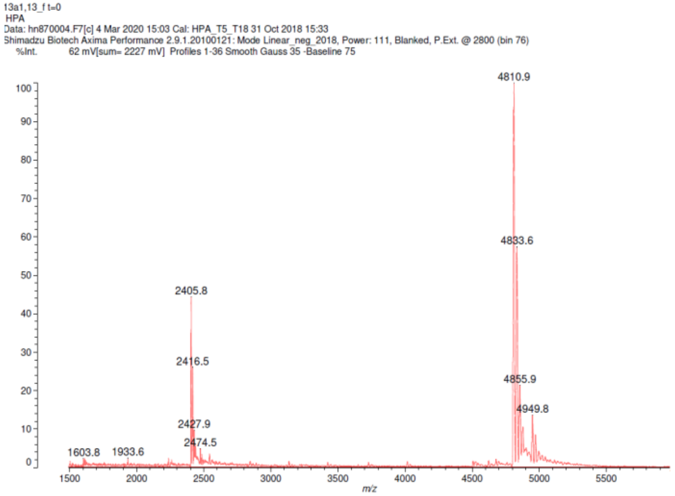

2 5'-d(CTC CAG AGC CCG A)-3'

(*)

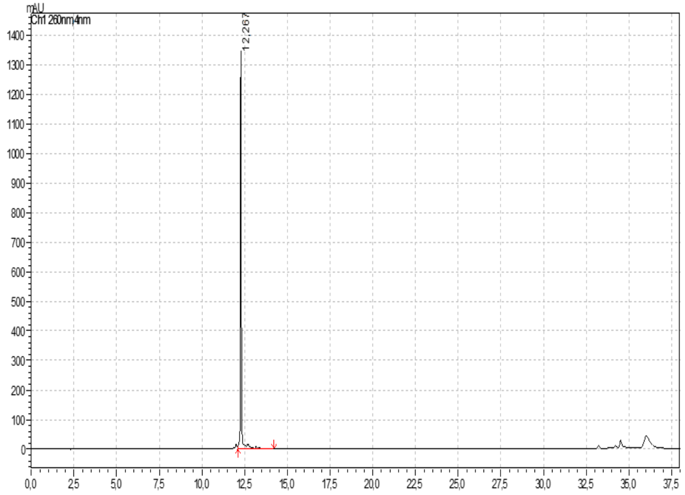

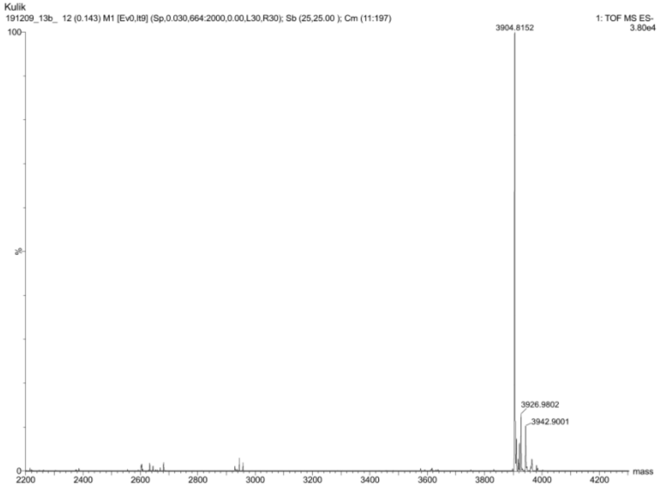

P2a 5'-d(C**P**C CAG AGC CCGA)-3'

(*)

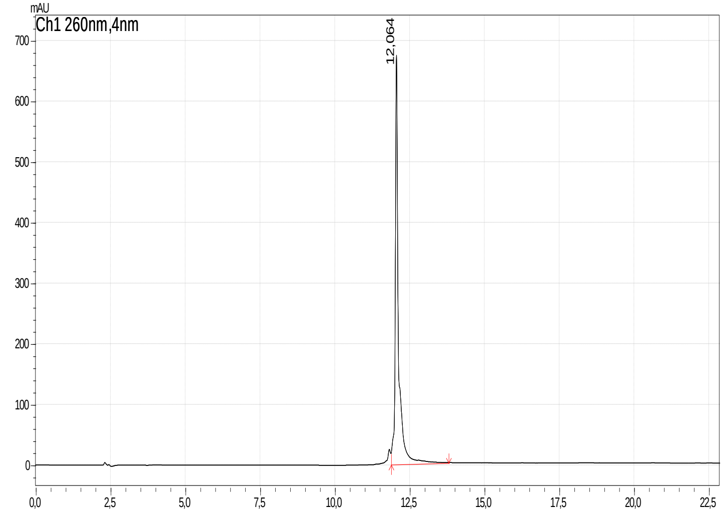

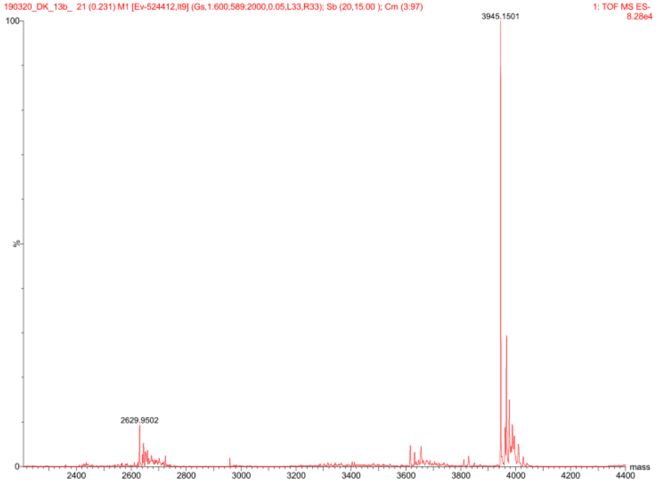

2a 5'-d(C**B**C CAG AGC CCGA)-3'

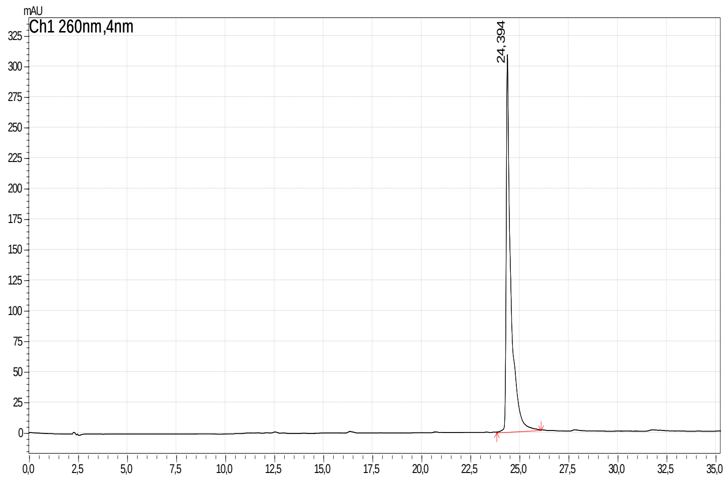

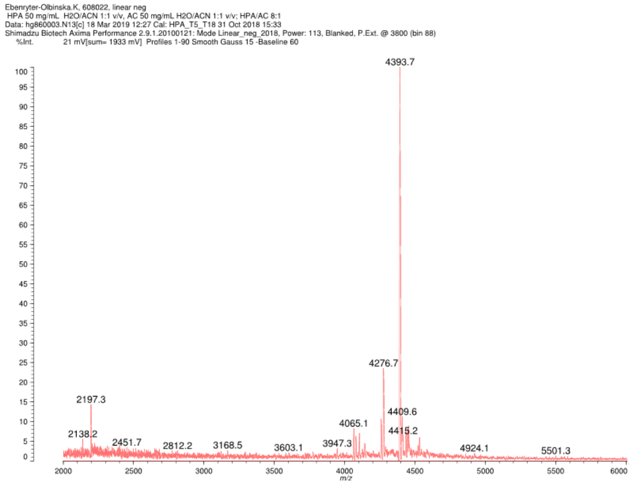

P2b 5’-d(CTC CAG AGC C**P**GA)-3’

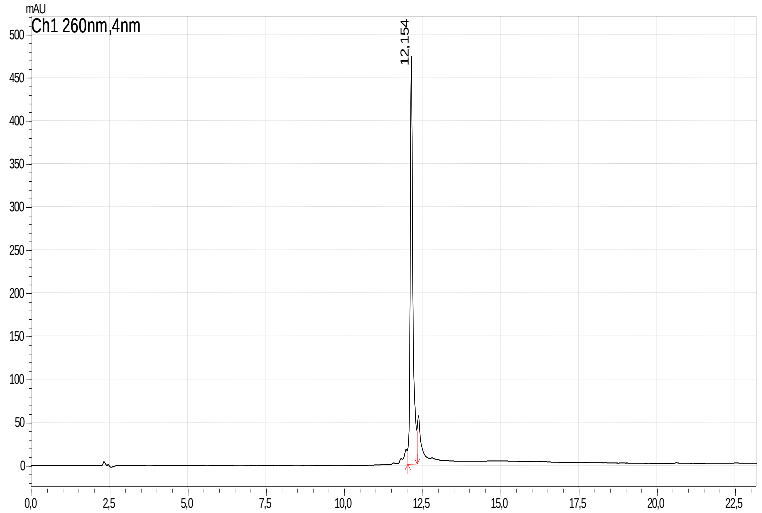

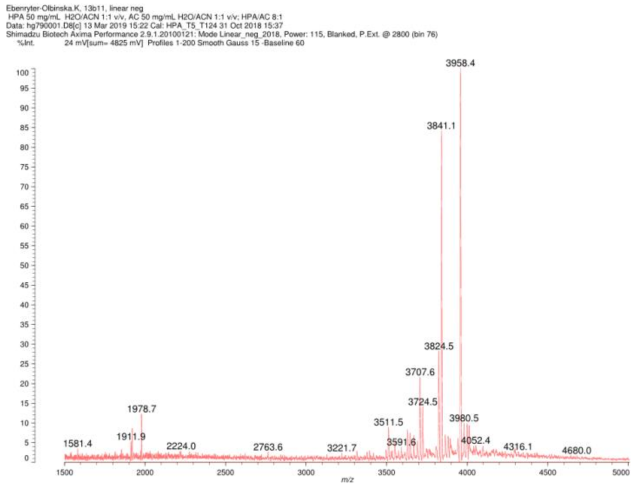

2b 5’-d(CTC CAG AGC C**B**GA)-3’

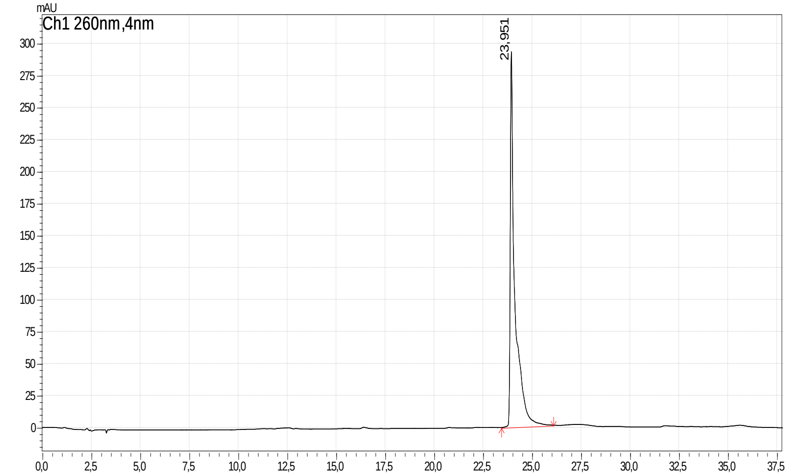

(*)

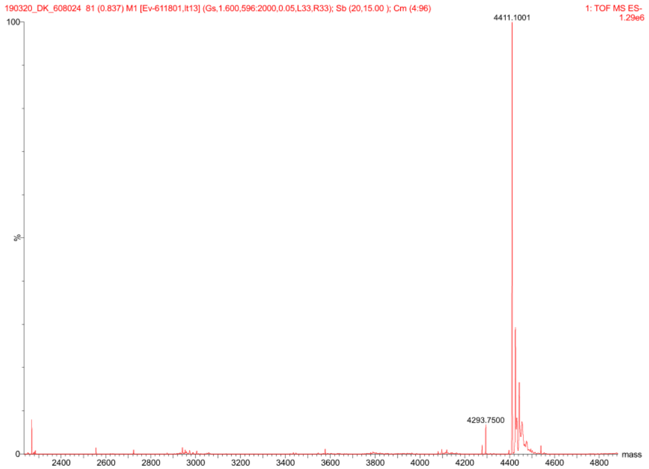

FL-1 5’-6FAM-d(TTT CTT TTC CTC C)-3’

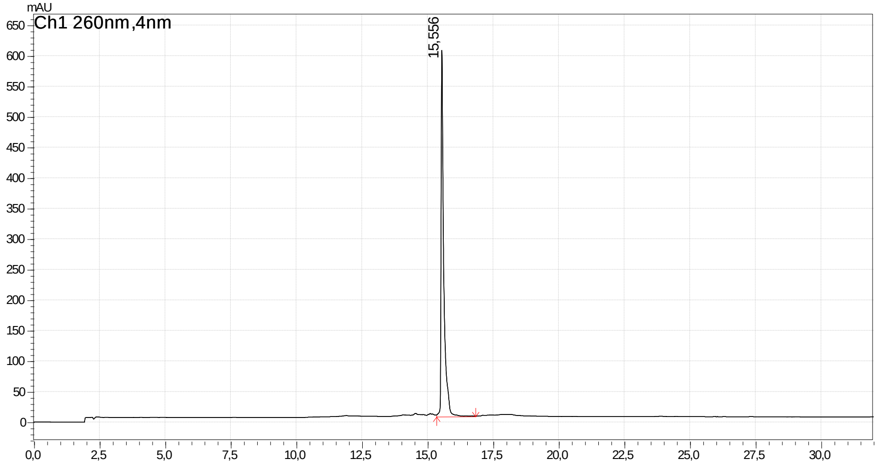

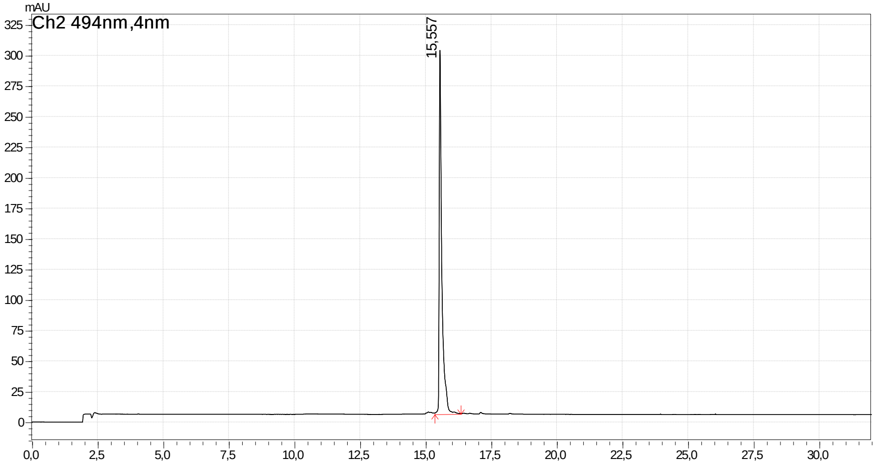

(*)

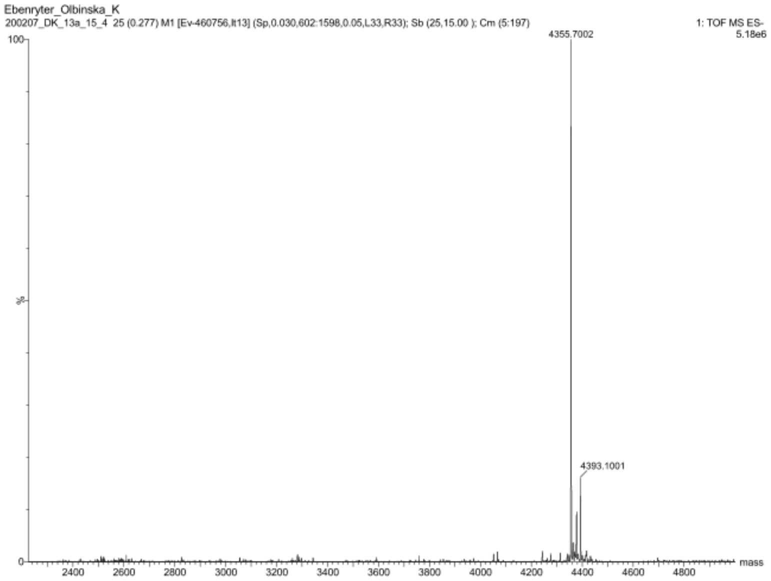
Ebenryter_Olbinska_K
200207_DK_13a_15_4 25 (0.277) M1 [Ev-460756,It13] (Sp,0.030,602:1598,0.05,L33,R33); Sb (25,15.00); Cm (5:197)
1: TOF MS ES-
5.18e6
4355.7002
4393.1001
100
%
0
mass
2400
2600
2800
3000
3200
3400
3600
3800
4000
4200
4400
4600
4800

FL-2 5’-6FAM-d(CTC CAG AGC CCG A)-3’

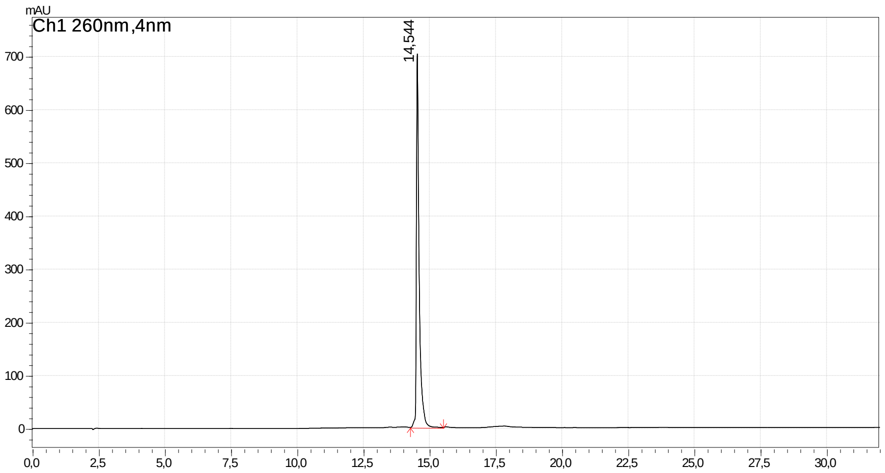

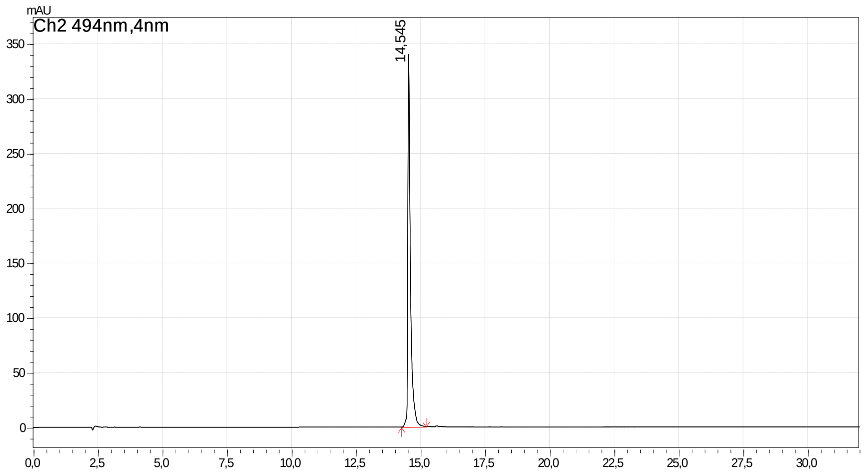

(*)

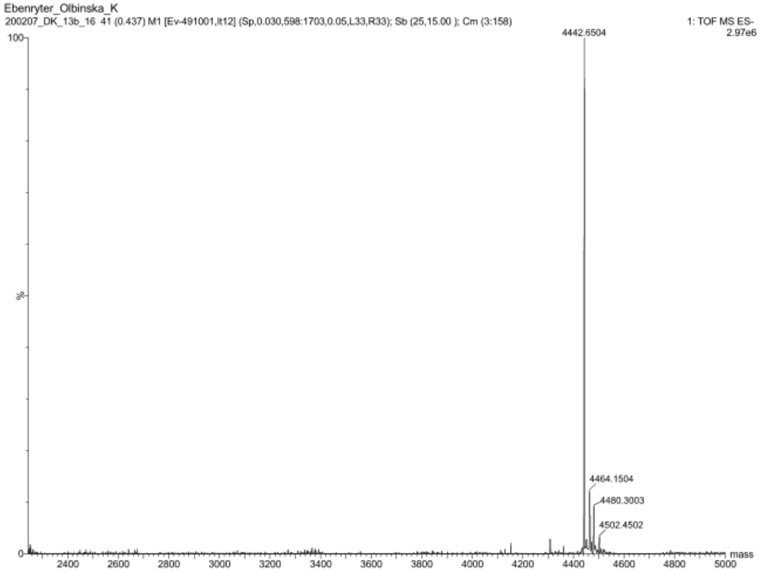

Figure S2. PAGE analysis of the **1, 1a, 1b, 1c** and **2, 2a, 2b** oligonucleotides (20%/7 M urea)

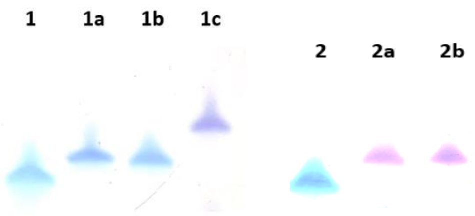

Figure S3. svPDE - assisted hydrolysis of reference oligonucleotide **1** with and without magnesium ions, monitored by MALDI-TOF mass spectrometry.

**1** (no $MgCl_2$)<br>negative control	**1** + $MgCl_2$<br>positive control
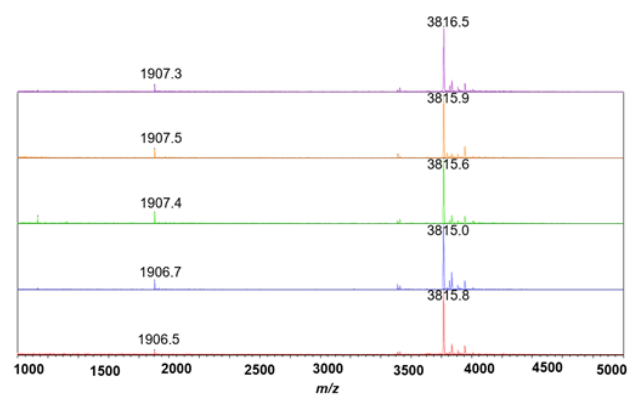	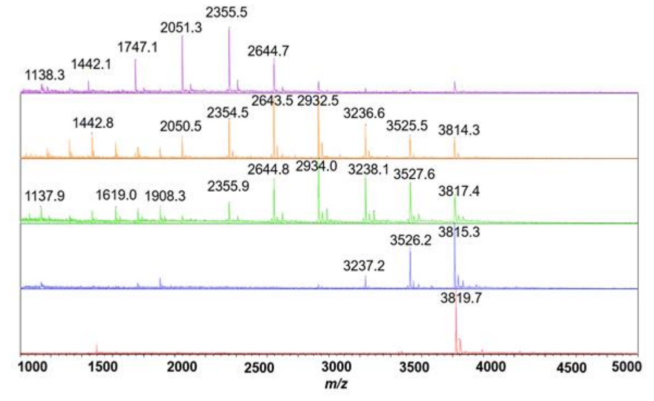

Figure S4. Original MALDI-TOF MS spectra of hydrolysis of **1c** in the presence of svPDE (in triplicate)

i) First experiment

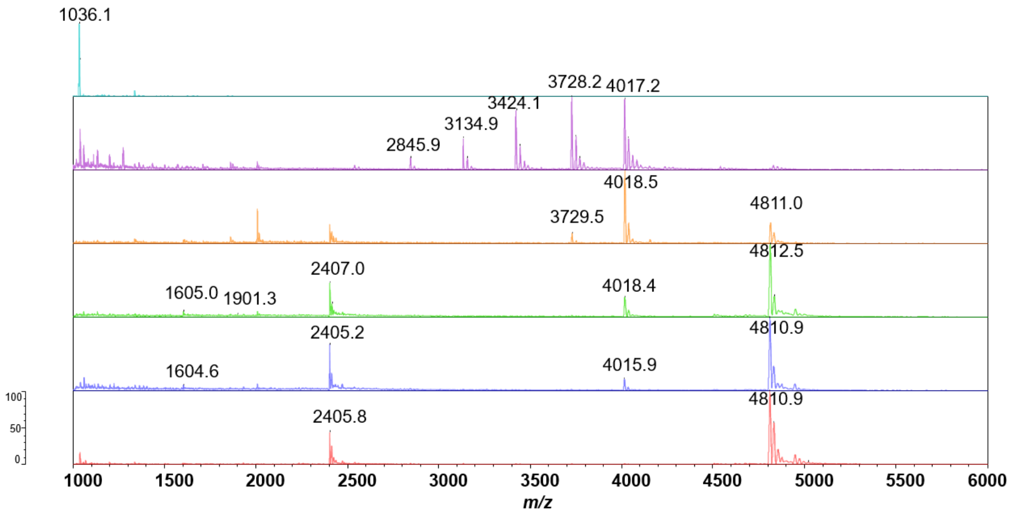

ii) second experiment

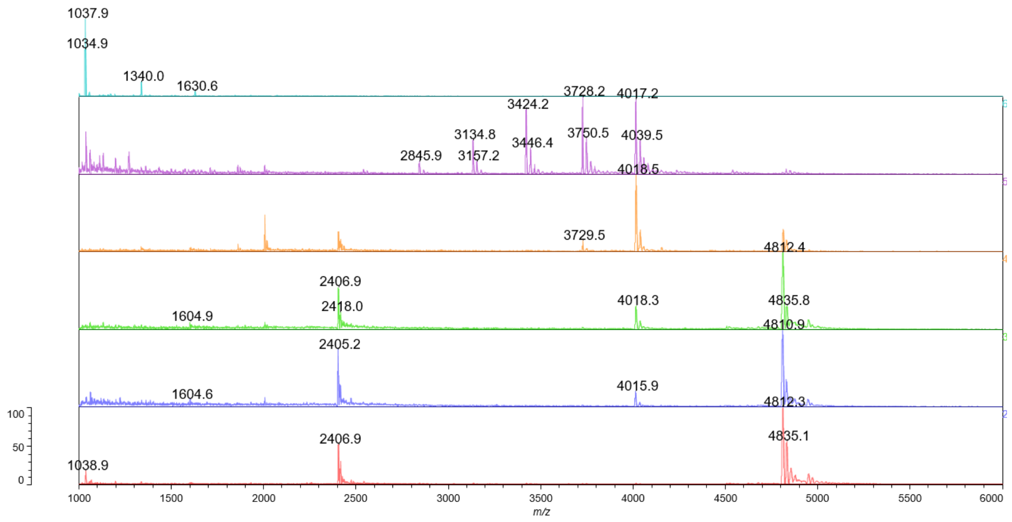

iii) third experiment

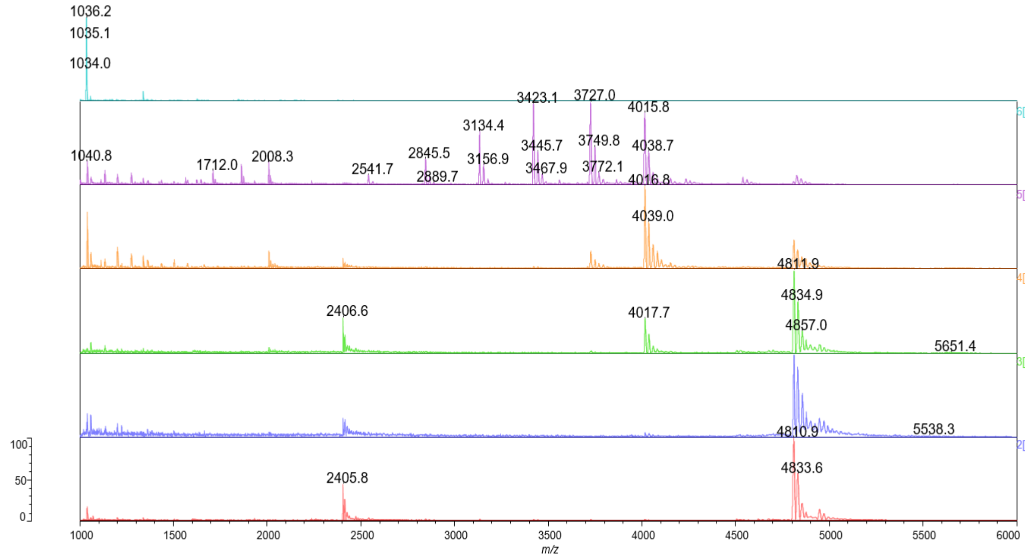

Figure S5. MALDI-TOF mass spectrometry analysis of the 1:1:1 mixture of **1, 1a** and oligonucleotide **1** containing two FESAN clusters (synthetic data not shown). Intensity of peaks is given on the Figure.

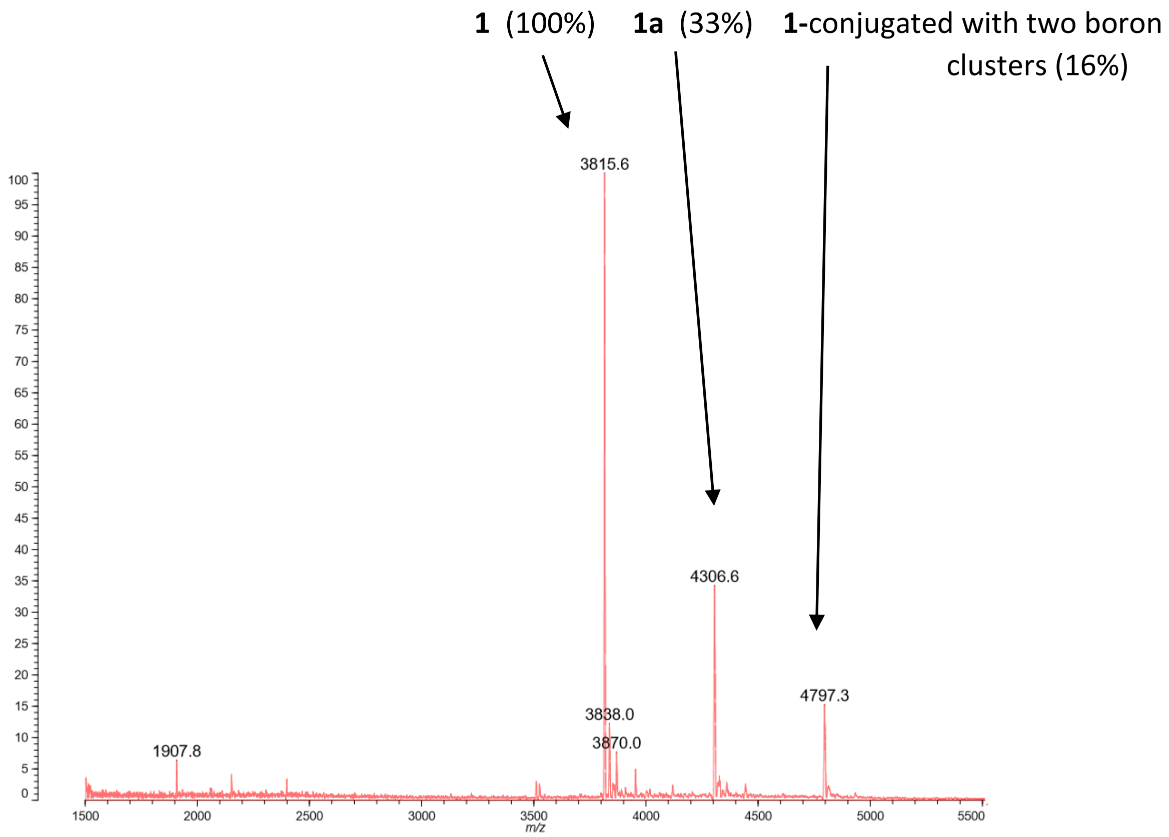

Figure S6. Figure S6. Original MALDI-TOF MS spectra of the hydrolysis mixture of **1+1a** in the presence of svPDE (in triplicate).

first experiment

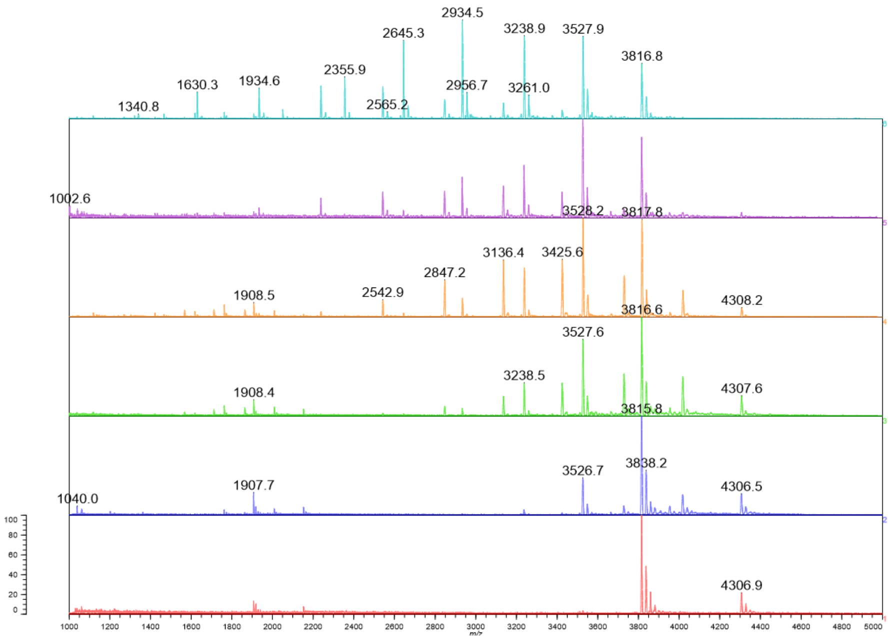

i) second experiment

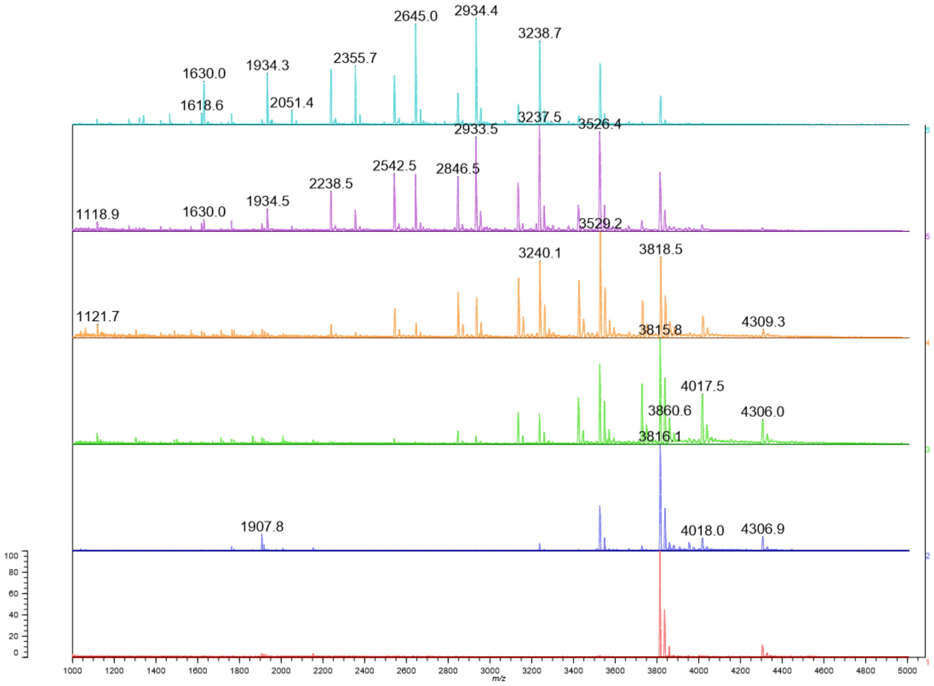

ii) third experiment

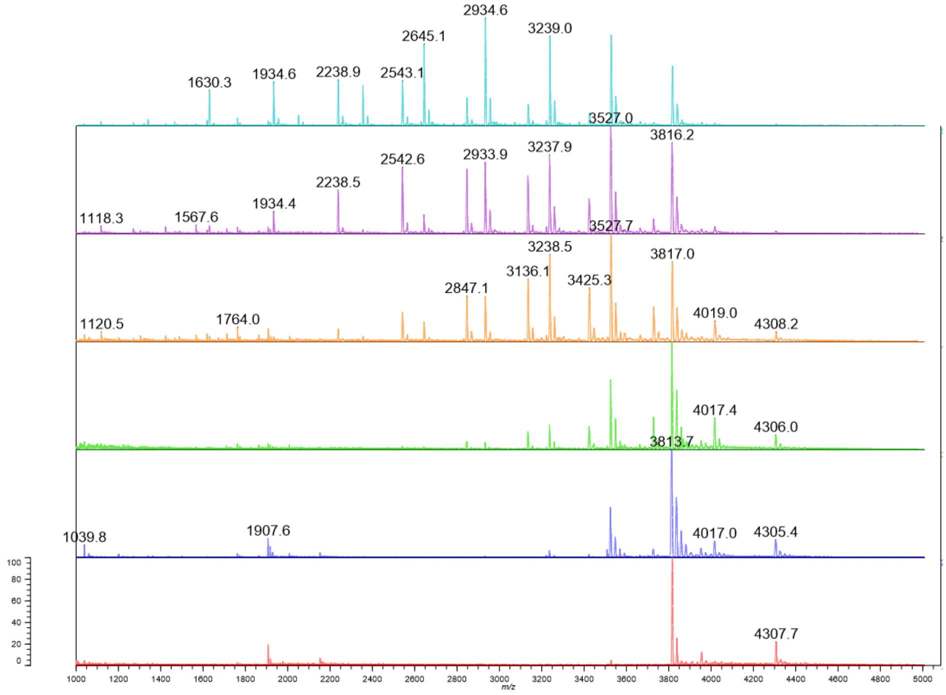

Figure S7. MALDI-TOF MS analysis of the reaction mixture of svPDE-assisted hydrolysis of unmodified oligonucleotides **1** and **2** (0.1 OD) in the presence of a free metallacarborane (FESAN, 182 nM) at 0-60 min.

**1 + FESAN**	**2 + FESAN**
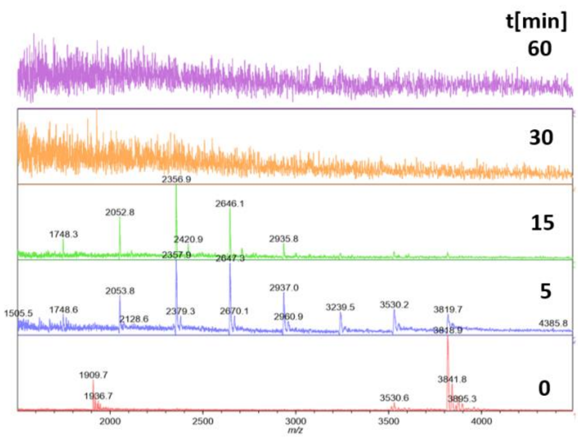	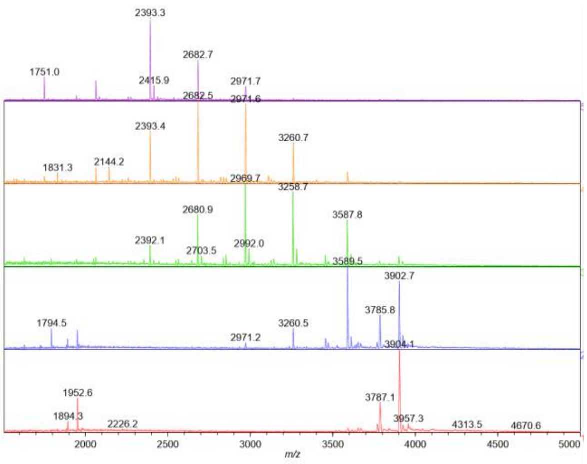

Figure S8. MALDI-TOF analysis of the hydrolysis rate of oligonucleotide **1** in the svPDE-assisted reaction carried out in the presence of 0, 1, 2 or 3 equivalents of metallacarborane (in triplicate)

i) measurements at 0 min.

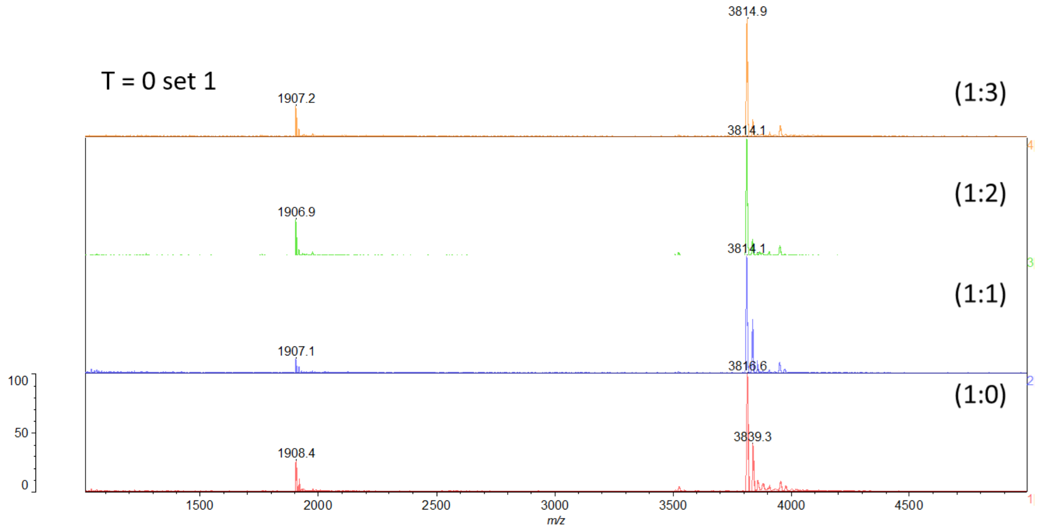

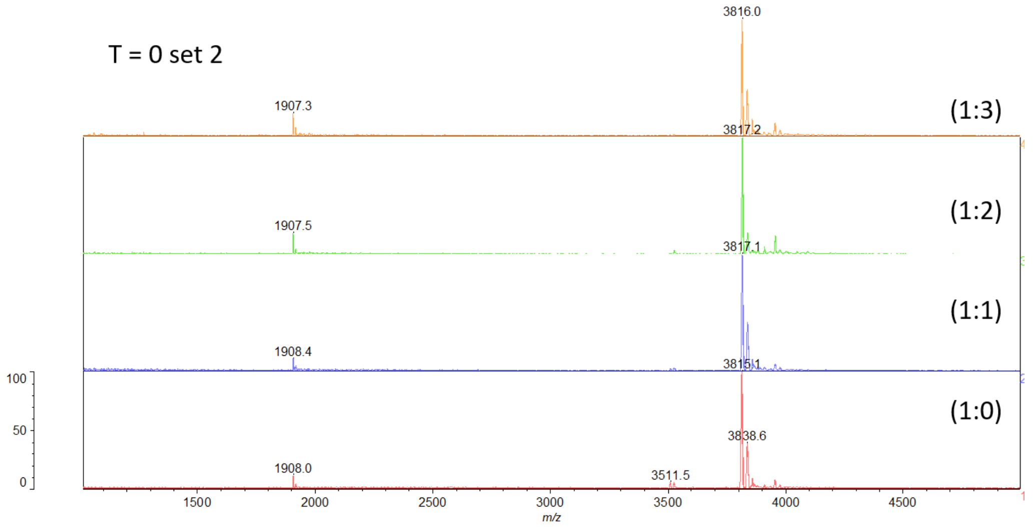

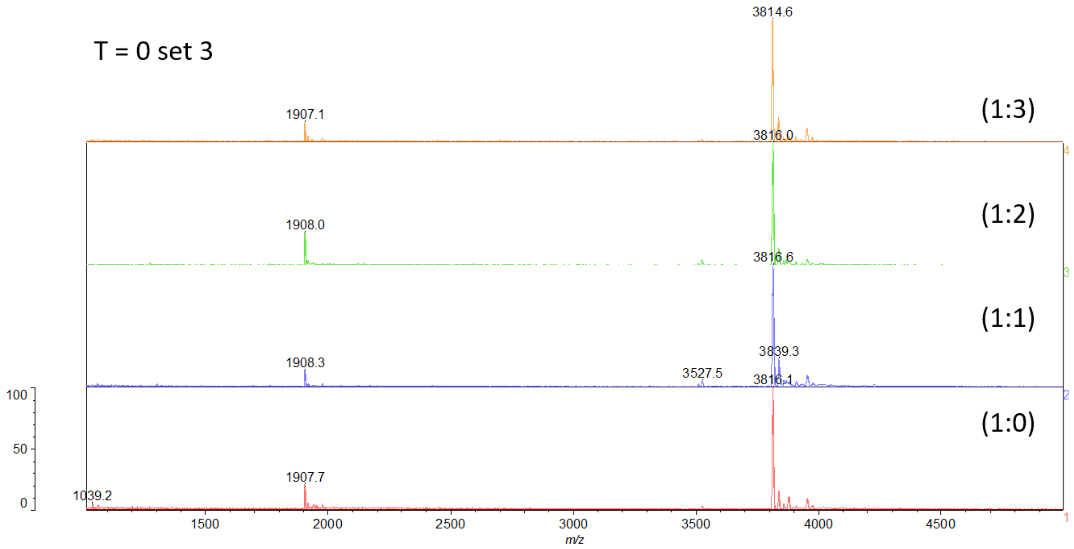

ii) Measurements at 5 min.

T = 5 set 1

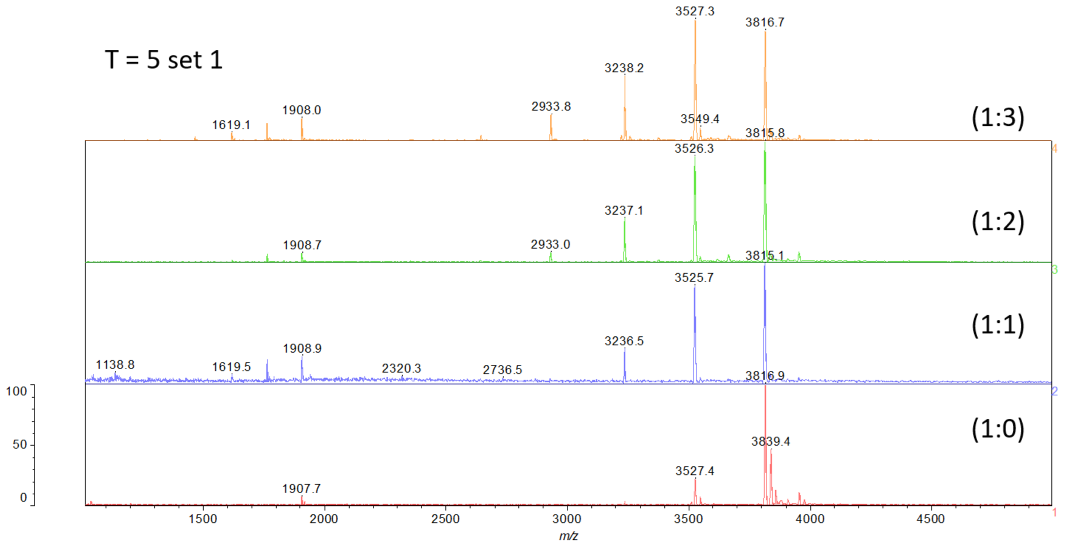

T = 5 set 2

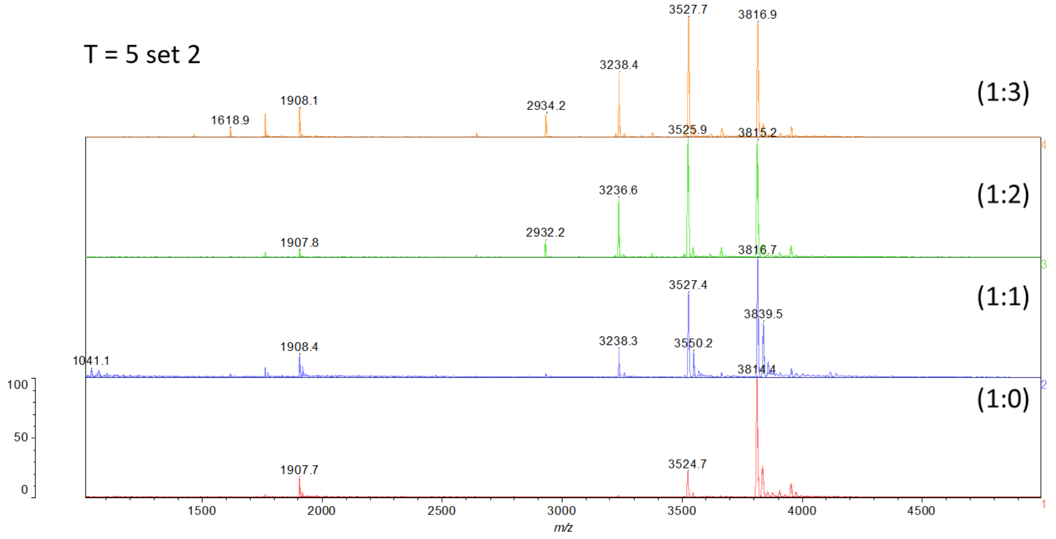

T = 5 set 3

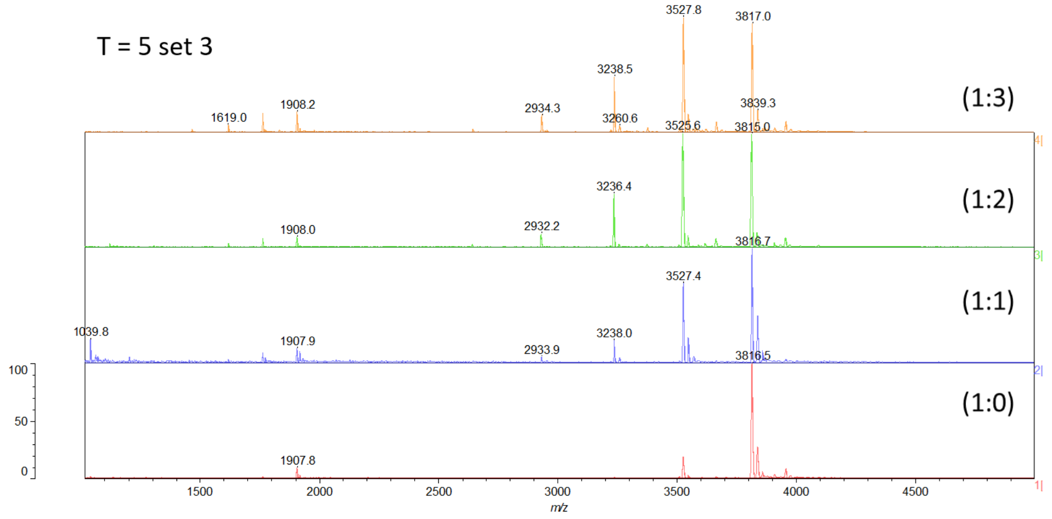

iii) Measurements at 15 min.

T = 15 set 1

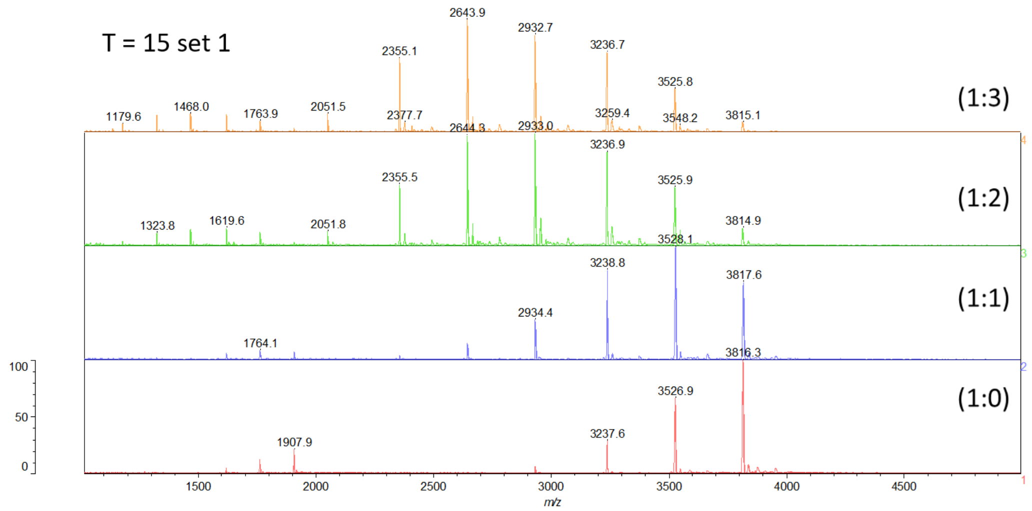

T = 15 set 2

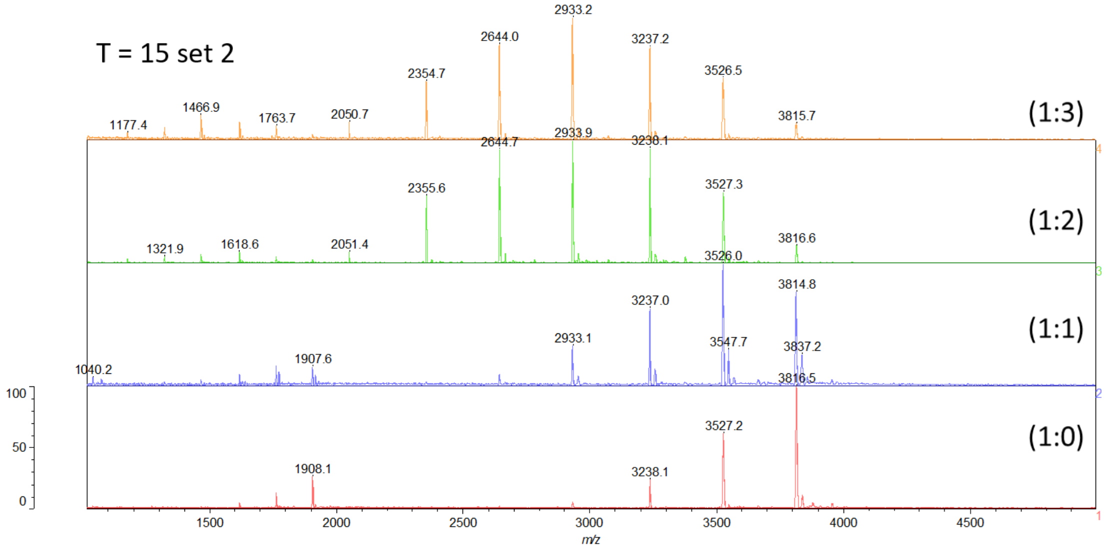

T = 15 set 3

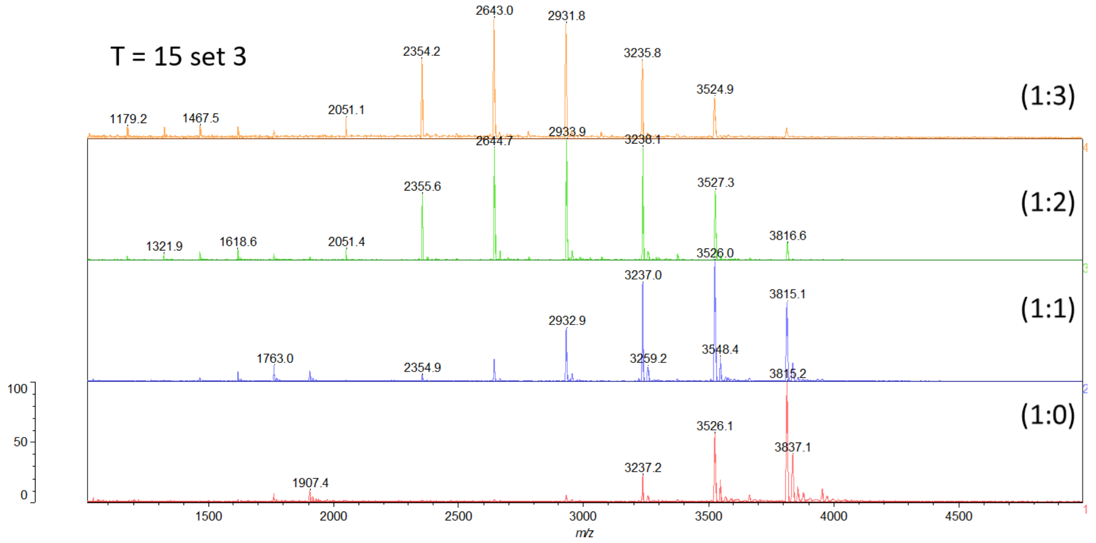

Figure S9. PAGE analysis of crude snake venom.
Lane 1 – molecular weight lader,
lane 2 – the sample of crude svPDE of MW 112 kDa (marked).

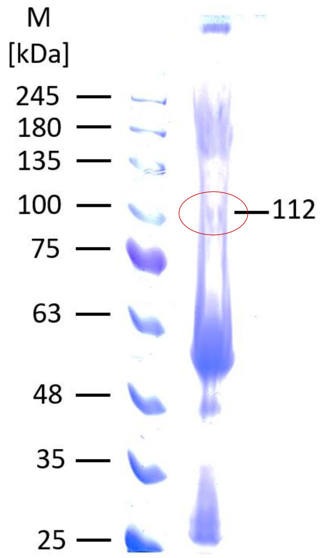

Figure S10. The protein samples capillary scan from microscale thermophoresis (the venom proteins fluorescently labeled with RED-NHS 2nd generation).

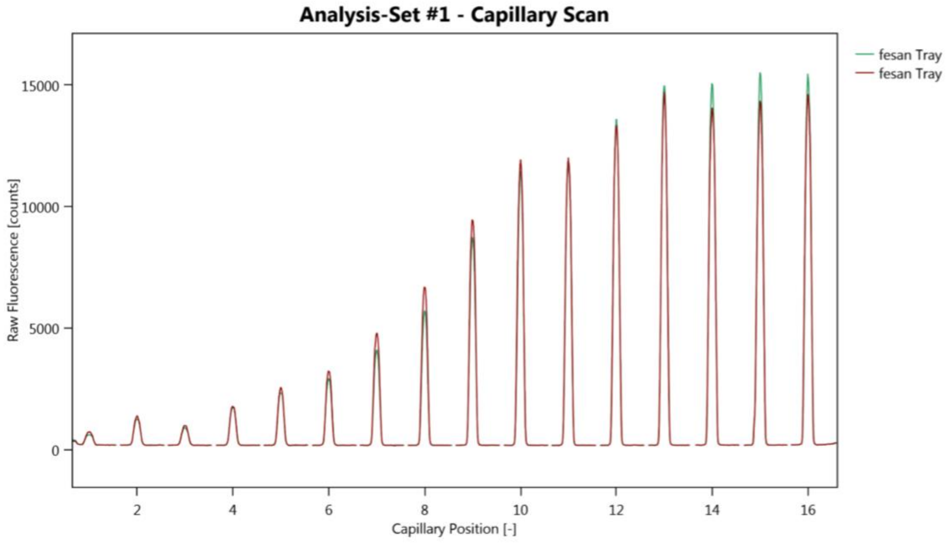

Figure S11. MST data for K_d determination of affinity of FESAN (ferra(III) bis(dicarbollide)) to **FL-1** (K_d=5.48±0.20 μM).

**Sample Information**	
**Merge-Set Name:**	Fesan Tray
**Date of Measurement:**	2020-02-07 11:51:19.022 → 2020-02-07 15:36:37.773
**Capillary Type:**	Monolith NT.115 Standard Treated Capillary
**Target:**	1-FL
**TargetConcentration:**	0,00000001
**Ligand:**	Fesan
**Measurement Settings**	
**MST-Power:**	Medium
**Excitation-Power:**	20%
**Excitation type:**	Green
**Thermostat Setpoint:**	22,0°C
**Fit Results (Kd)**	
**Fit Model:**	Kd
**Bound**	903,4445704
**Unbound**	~~923,7399589~~
**Kd**	5,4834E-06
**TargetConc**	~~0,00000001~~
**Standard Deviation:**	0,207569137
**Kd Confidence:**	±4,34262E-06
